# Early childhood stress responses to psychosocial stressors: The state of the science

**DOI:** 10.1002/dev.22320

**Published:** 2022-09-05

**Authors:** Randi A. Bates, Lisa Militello, Erin Barker, Hugo Gonzalez Villasanti, Kammi Schmeer

**Affiliations:** ^1^ College of Nursing University of Cincinnati Cincinnati Ohio USA; ^2^ College of Nursing The Ohio State University Columbus Ohio USA; ^3^ Crane Center for Early Childhood Research and Policy The Ohio State University Columbus Ohio USA; ^4^ Department of Educational Studies, College of Education and Human Ecology The Ohio State University Columbus Ohio USA; ^5^ Department of Sociology The Ohio State University Columbus Ohio USA

**Keywords:** autonomic nervous system, child, parent–child relations, pituitary adrenal system, stress

## Abstract

The aim of this systematic review was to better understand whether and to what extent psychosocial stressors are associated with hypothalamic–pituitary–adrenal axis or autonomic nervous system stress responses in young children (1–6 years of age). Studies were classified by psychosocial stressors from the ecobiodevelopmental model: social and economic resources, maternal mental health, parent–child relationships, and the physical environment. Of the 2388 identified studies, 32 met full inclusion criteria, including over 9107 children. Child physiologic stress responses were measured as hair and urinary cortisol and cortisone, salivary diurnal and reactive cortisol, salivary reactive alpha‐amylase, and respiratory sinus arrhythmia. There were 107 identified relations between psychosocial stressors and physiologic stress responses. Nearly two thirds of these relations suggested that children have dysregulated stress responses as either significantly blunted (*n* = 27) or increased (*n* = 37); 43 relations were not significant. Children most consistently had significantly dysregulated stress responses if they experienced postnatal maternal depression or anxiety. Some reasons for the mixed findings may be related to characteristics of the child (i.e., moderators) or stressor, how the stress response or psychosocial stressor was measured, unmeasured variables (e.g., caregiving buffering), researcher degrees of freedom, or publication bias.

## 1 INTRODUCTION

Experiencing adverse psychosocial stressors in early childhood can have profound implications on lifelong public health. An adverse psychosocial stressor is a life situation that may create an intense level of stress and may contribute to maladaptive behavior, illness, or a mental disorder later in life (American Psychological Association, 2020). Research estimates suggest that psychosocial stressors, such adverse childhood experiences (ACEs), cost the United States nearly $750 billion/year (Bellis et al., 2019). These costs are due to the deleterious effects of ACEs on maladaptive health behaviors, resulting in chronic disease (Bellis et al., 2019).

Young children regulate stress responses to perceived psychosocial stressors by physiologically adapting to their environment (Blair et al., 2011). A young child, defined here as between approximately 1 and 6 years of age, is undergoing a particularly sensitive developmental period when brain circuitry begins to selectively prune to optimally fit the child's lived environment (for a brief review, see Sakai, 2020)—such as encountering environmental stressors. Also, during this period, young children undergo rapid development, building a robust socioemotional foundation in accordance with environmental experiences that will serve them through life (e.g., Moffitt et al., 2011).

Adverse psychosocial stressors of significant concern are those most proximal to the young child, occurring in family‐level contexts (Bronfenbrenner & Morris, 2006). The ecobiodevelopmental framework proposes that family‐level stressors are related to adverse caregiver resources, family relationships, and the physical environment (Shonkoff, 2012). Adverse caregiver resources could include limited resources that make it challenging to provide the optimal conditions to raise a young child, such as low socioeconomic status, poor mental health, and food insecurity. Socioeconomic resources may also be broad and include parental resources such as income, education, race/ethnicity, employment, and housing. Caregiver age may also be a socioeconomic resource. Having a lower age as a parent, particularly as a teenager, is associated with less experience, education, and resources to support a young child compared to older parents. Adverse family relationships may include psychosocial stressors such as hostile parenting or neglect that fail to provide young children with consistent nurturing and protective interactions that promote optimally regulated stress response systems. Unsafe environments may be those not free from toxins, fear, or risk of harm, or those limiting the child's active exploration necessary for development. Such stressors may directly provoke physiological stress responses along the hypothalamic–pituitary–adrenal (HPA) axis and the autonomic nervous system (ANS) (Shonkoff, 2010; Shonkoff et al., 2012). Experiencing intense or prolonged stress responses may influence the development of health behaviors and chronic disease due to the child physiologically adjusting and adapting to these stress responses during sensitive periods of development (Shonkoff, 2010; Shonkoff et al., 2012). Yet, this adaptation helps the child manage survival within their current environment (Blair et al., 2011).

The ecobiodevelopmental framework proposes three general child stress responses to stressors that result in “stress”—including positive, tolerable, and toxic stress—based on the long‐term consequences for child development (Shonkoff et al., 2012). Positive stress results when a child experiences a brief physiologic stress response that is mild to moderate in magnitude (Shonkoff et al., 2012). For example, a child's anxiety associated with experiencing their first day at an early educational center (e.g., preschool or daycare) could induce positive stress. If a child receives “buffering” from an adult caregiver (e.g., love, support), positive stress can promote the child's growth and advancement along the course of the child's development (Shonkoff et al., 2012). Tolerable stress results from a child's exposure to nonnormative experiences (e.g., divorce, family member death). Tolerable stress could lead to excessive or prolonged activation of the stress response system, but buffering from an adult can reduce the risk of prolonged stress response activation and long‐term consequences on the child's health. The third and most dangerous type of stress is toxic stress; examples are experiencing child abuse, neglect, or maternal depression (Shonkoff et al., 2012). Toxic stress is theorized to inappropriately disrupt the normal development of brain circuitry and other crucial organs during early childhood (Shonkoff et al., 2012), resulting in adverse health outcomes (Blair & Raver, 2012).

### 1.1 Physiological Stress Responses in Children

In young children, stress responses are thought to most immediately occur along the ANS or the HPA axis (for a review, see Condon, 2018). The ANS is primarily responsible for controlling the immediate fight‐or‐flight response to stress. Three common measures of the ANS are heart rate variability (HRV), respiratory sinus arrhythmia (RSA), and salivary alpha‐amylase. The HPA axis is involved in regulating diurnal physiology and rapid responses to perceived stress that direct resources to help an organism survive. A common measure of stress from the HPA axis is cortisol. Cortisol can be measured in saliva, serum, urine, hair, and nails. In young children, researchers traditionally measured cortisol in saliva, but more recently in hair (Bates et al., 2017). At this time, there is no “gold‐standard” way of measuring stress responses in young children. Each biomarker of stress may have its advantages and disadvantages in approximating stress responses, as these biomarkers may also concurrently reflect ongoing processes to support normal physiologic processes. Additional details of measures of the ANS (including HRV, RSA, and alpha‐amylase) and HPA axis (including cortisol and cortisone) are discussed next.

#### 1.1.1 Autonomic Nervous System (ANS)

The ANS is primarily responsible for controlling the immediate fight‐or‐flight response to stress. The ANS can be divided into two physiological response systems, the sympathetic nervous system (SNS) and the parasympathetic nervous system (PNS). The SNS is responsible for preparing and mobilizing the body during stress, producing a heightened state of arousal characterized by increased heart rate, increased blood pressure, pupil dilation, inhibition of the digestive and urinary systems, and sweating. To balance the SNS stress response, the PNS supports a state of rest and repair that is characterized by decreased blood pressure, reduced heart rate, and digestive and urinary system stimulation, allowing the body time for restoration of energy stores. Three common measures of the ANS are HRV, RSA, and alpha‐amylase. Depending on how these ANS measures are collected, they may reflect SNS or PNS activation or interplays.

##### Heart Rate Variability (HRV)

HRV can be used as a biomarker to assess the stress response of the ANS, as it is modulated by both SNS and PNS activity (Ernst, 2017; Kleiger et al., 1987). HRV is the dispersion in the time intervals between adjacent heartbeats (R–R interval), also known as interbeat intervals (IBI). The IBI signal is detected from electrocardiogram or photoplethysmogram (PPG) sensors using wave detection algorithms (Tarvainen et al., 2014). HRV measures can be classified into time‐domain, frequency‐domain, and nonlinear characteristics to reflect properties of heart rate dynamics (Shaffer & Ginsberg, 2017). For reference, the approximate median standard deviation of the IBI of normal sinus beats (SDNN, time‐domain) for preschoolers ages 3–5 years is approximately 95 ms (van den Berg et al., 2018). Decreased HRV is usually associated with pathology and poorer prognosis, while positive health outcomes have been noted in those with varied HRV (Thayer et al., 2010; Tsuji et al., 1994). In other words, decreased HRV is generally associated with higher levels of stress (Jarczok et al., 2013; Michels et al., 2013).

##### Respiratory Sinus Arrhythmia (RSA)

RSA is another useful biomarker of the ANS, particularly PNS mediation of the heart's electroconductivity (Beauchaine, 2015). RSA is assessed by the variability in an individual's heart rate across a respiratory cycle (Ravindran et al., 2021). During the respiratory cycle, the heart's normal electroconductivity of the R–R interval is shortened during inspiration and lengthened during expiration (Yasuma & Hayano, 2004). When stress is minimal, increased resting RSA (in other words, higher or more variability) and a decrease in RSA during stress (in other words, RSA augmentation or increased vagal nerve control over the heart, resulting in decreased PNS and RSA reactivity; Zhang et al., 2017) is associated with better emotion regulation related to stressful experiences (Gouin et al., 2014; Park & Thayer, 2014). Conversely, decreased resting RSA (in other words, lower or less variability) and a reactive increase in RSA in response to stress (in other words, RSA withdrawal or suppression, indicating withdrawal of the vagus nerve control over the heart and increased PNS reactivity; Zhang et al., 2017) is associated with several stress‐related disorders such as anxiety, depression, and internalizing and externalizing symptoms (for a review, see Beauchaine, 2015). In some instances, increased RSA reactivity is beneficial in increasing alertness for particular tasks; however, it may also lead to increased expression of emotions (Zhang et al., 2017).

##### Alpha Amylase

Alpha‐amylase is a salivary enzyme primarily involved in digesting starch found in the oral cavity, but has been increasingly recognized as a useful biomarker of SNS stress responses (Nater & Rohleder, 2009). Salivary alpha‐amylase increases within 2–10 min after a sympathetic response to a perceived stressor (Davis & Granger, 2009), with recovery time about 10–20 min later (Engert et al., 2011). In contrast, PNS stimulation lowers salivary alpha‐amylase concentrations (Anderson et al., 1984; Engert et al., 2011).

#### 1.1.2 Hypothalamus‐Pituitary‐Adrenal (HPA) axis

The HPA axis is involved in regulating diurnal physiology and rapid responses to perceived stress to direct more resources to help an organism survive, such as fighting or fleeing a stressor. In response to a stressor, the hypothalamus releases corticotropin‐releasing factor and arginine vasopressor toward the pituitary gland. Upon receiving these neurohormone messengers, the pituitary gland produces and secretes adrenocorticotropic hormone into the bloodstream, which signals the adrenal glands to produce and release glucocorticoid hormones. In humans, this main glucocorticoid is cortisol (in rodents, corticosterone). Cortisol is sometimes involved in feedback loops to the hypothalamus or is physiologically converted into an inactive metabolite, cortisone.

##### Cortisol and Cortisone

Because of its role in fight or flight responses to stress, cortisol is often considered a major “stress hormone” and is consequently measured in several research studies on stress. Cortisol can be measured in saliva, serum, urine, hair, and nails. In young children, researchers typically measure cortisol in saliva and more recently, in hair (Bates et al., 2017). Samples of salivary cortisol can reflect diurnal pulsations of cortisol (with typical increases after awakening and then decreases through the day) or reactivity toward stressors: cortisol typically increases about 20 min after a perceived stressor with a recovery of about 70 min later (Engert et al., 2011). There are several ways to calculate salivary cortisol stress responses: measures can reflect basal averages, awakening responses, total hormone concentration, or varied ways to calculate changes over time (Pruessner et al., 2003). Hair cortisol typically reflects the average amount of unbound cortisol expressed in the body over at least 1 month of time. Researchers have shown that both high and low levels of cortisol may reflect significant stress, with low levels perhaps reflecting more chronic levels or clinical diagnoses of stress (Xu et al., 2019). Researchers have also begun measuring cortisone, and this is typically measured in young children in hair or urine. Researchers theorize that because cortisone is a metabolite of inactive cortisol, it may be a more accurate marker of serum cortisol (Blair et al., 2017).

### 1.2 Research Gaps

Several reviews have contributed to our understanding of individual markers of stress in children (e.g., Bates et al., 2017; Bryson et al., 2021; Davis et al., 2018; Hunter et al., 2011; Schär et al., 2022; Soares et al., 2021). Findings from these reviews strongly suggest that adverse psychosocial experiences at an early age are associated with biological risk and the development of disease through alterations in physiology. However, there is a lack of evidence as to how early childhood stress responses may be environmentally primed. The Bates et al. (2017) and Bryson et al. (2021) reviews only examined hair cortisol concentration as a marker of physiological stress, which captures some aspects of chronic (i.e., 1 month or more) physiological stress plus cortisol concentrations needed for typical physiological functions. Similarly, Hunter et al. (2011) only focused on studies that examined salivary cortisol responses to stressors. The Soares et al. (2021) review focused on longer term implications of experiencing more severe ACEs throughout childhood (up to 18 years old), such as markers of biological embedding, including effects on the immune system, structural and functional brain changes, and genetic and epigenetic markers. The review also included only 10 studies (out of 58) of children ages 1–6 years of age, with no studies on children below 3 years old. Finally, the Schär et al. (2022) study only examined pediatric maltreatment and HPA axis responses. There are also three other known meta‐analyses on adversity and stress responses, but these analyses were limited to measures of cortisol and sometimes included adult participants (Bernard et al., 2017; Bunea et al., 2017; Fogelman & Canli, 2018). Hence, there is a need to summarize the current literature on how young children may produce stress responses to psychosocial stressors, particularly along the immediate acting HPA axis and ANS.

### 1.3 The Current Study/Review

The aim of this review was to better understand whether and to what extent psychosocial stressors are associated with HPA axis or ANS stress responses in young children (1–6 years of age). We focused on children 1–6 years of age, as these children likely developed some psychomotor independence beyond infancy and are in a rapid period of developmental socioemotional growth that may be particularly sensitive to psychosocial stressors. Additionally, as suggested by ecological theories (Bronfenbrenner & Morris, 2006; Shonkoff, 2010; Shonkoff et al., 2012), we focused on family‐level psychosocial stressors that may have the most substantial influence on stress responses, compared to community‐level or policy‐level stressors. Lastly, we carefully outline both heightened and blunted stress responses in young children. A heightened stress response indicates that the stress response was increased and blunted means that the stress response was dampened. Both heightened and blunted stress responses may be significant indicators of stress responses (Ford et al., 2019; Khoury et al., 2019; Miller et al., 2007; Xu et al., 2019) and highlight potential physiological disruptions during sensitive periods of development.

## 2 METHODS

### 2.1 Protocol and Registration

The protocol for this review was developed a priori and submitted to PROSPERO in December 2019 (Appendix SA). This systematic review was guided by the Preferred Reporting Items for Systematic Reviews and Meta‐Analysis Protocol (PRISMA‐P; Moher et al., 2015).

### 2.2 Inclusion and Exclusion Criteria

Inclusion criteria were as follows: peer‐reviewed empirical studies published through September 18, 2020, in English, observational or experimental in nature, and with neurotypical children ages 12–72 months. A study was included if the mean age range was within the target range (12–72 months), even if a study included a child that was greater than 72 months. Studies needed to include a family‐level psychosocial stressor, as outlined by the ecobiodevelopmental framework (Shonkoff, 2010; Shonkoff et al., 2012), as the independent variable. Psychosocial stressors were categorized following the ecobiodevelopmental framework, specifically: caregiver resources (e.g., socioeconomic status [SES] and mental health), child and family relationships (e.g., parenting, family functioning, child maltreatment, family violence), and the physical environment (e.g., household safety). Additionally, studies had to report on the child's stress response from the HPA axis (e.g., cortisol or cortisone) or the ANS (e.g., RSA, HRV, or salivary alpha‐amylase) as the dependent variable.

Exclusion criteria were as follows. Studies concentrating on a specific population of neurodivergent children (e.g., attention deficit hyperactivity disorder, prematurity of less than 37 weeks of age, known exposure to prenatal substance use disorders, a neurological medical condition associated with altered HPA axis or ANS functioning known at recruitment) were excluded due to potentially atypical stress responses. Studies were excluded if stress was induced without understanding its relationship to a psychosocial stressor (i.e., laboratory‐induced stressor to measure response in a controlled environment without understanding its relationship to a psychosocial stressor). We also excluded articles specifically focused on children with asthma or taking asthma‐related medications due to potential medication reporting confusion between taking a steroid and albuterol (Bates et al., 2020)—steroids may interfere with cortisol physiology (Gray et al., 2018). Lastly, reviews and non‐peer‐reviewed literature such as the gray literature, conference abstracts, editorials, and dissertations were excluded.

### 2.3 Search Strategy, Study Selection, and Data Extraction

Four databases—MEDLINE, EMBASE, PsycINFO, and CINAHL—were searched using the following search formula: “(child OR pediatric OR toddler OR preschool OR youth) AND (hypothalamus OR pituitary OR adrenal OR autonomic nervous system OR sympathetic nervous system OR parasympathetic nervous system) AND (stress OR distress).” Limits were set when applicable and included English language, peer‐reviewed journals, ages 0–6 years old, human subjects, and academic journals. Two authors (RAB, EB) first reviewed titles, then abstracts, then full texts to determine study eligibility. Discrepancies were resolved by discussion. Then, four authors (RAB, EB, LKM, HGV) reviewed the full text and independently extracted data using standardized criteria. These criteria included the study's original objective and type of study, characteristics of the sample (sample size, child ages, child race/ethnicity, child sex composition, and socioeconomic status), description of the psychosocial stressor in accordance with the ecobiodevelopmental model, the measured stress response, and the study's findings on the relations between measured psychosocial stressors and stress response. One author (RAB) verified all extracted information by validating back to the full text; further discrepancies were resolved by discussion with the author who originally extracted data from the article. Data were narratively synthesized and organized by type of psychosocial adversity. A meta‐analysis was not conducted due to the diverse methods and measures used across studies.

### 2.4 Appraisal of Study Rigor

Two reviewers (RAB, LKM) appraised the methodological quality of the studies using the Joanna Briggs Institute (JBI) critical appraisal tools (Moola et al., 2020; Tufanaru et al., 2020) to inform the synthesis and interpretation of the study results. The JBI tools were used due to the diverse study types included in this review (Higgins et al., 2011). Four JBI critical appraisal tools were used: case–control, cohort, cross‐sectional, and quasi‐experimental. Disagreements in study appraisal were resolved through discussion and with consensus by a third author (KS).

## 3 RESULTS

### 3.1 Search and Overview

**FIGURE 1:**
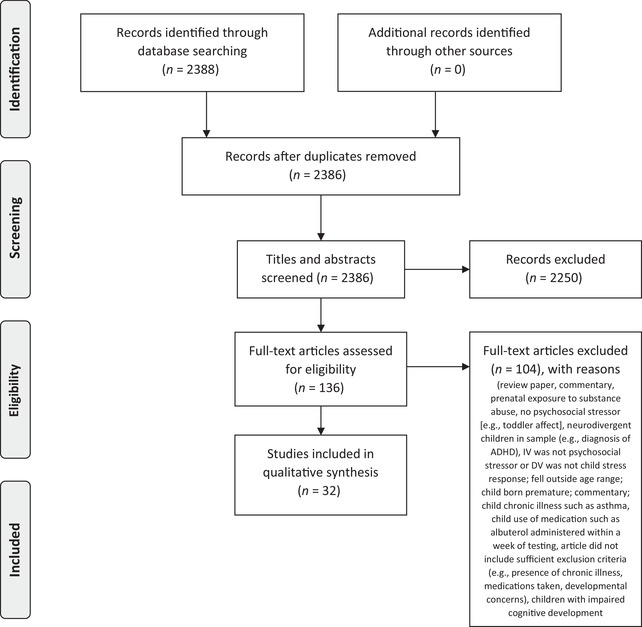
Study selection process based on PRISMA‐P guidelines. ADHD, attention deficit hyperactivity disorder; IV, independent variable; DV, dependent variable.

The results of the study search are summarized in Figure 1. The initial search generated 2388 studies; 32 articles remained for inclusion in this review. Study quality is highlighted in Table 1. The resulting studies with extracted characteristics and relationships are summarized in Table 2. Table 2 is organized by stress response (hair cortisol and/or cortisone, urinary cortisol and/or cortisone, diurnal salivary cortisol, reactive salivary cortisol, salivary alpha‐amylase, RSA) and then alphabetically by the first study author. Table 2 includes details on the study objective, the child characteristics (sample size, child age, child race/ethnicity, child sex, child SES), measures of the psychosocial stressor and stress response, and study findings. Tabulated categories of these characteristics—including child age, study country, psychosocial stressor, and stress response—by number of studies are shown in Table 3.

**TABLE 1:** Appraisal of studies

		Appraisal criteria by study type																	
		All studies								Case–control		Cohort	Cross‐sectional		Quasi‐experimental				
Study	Study type	Groups comparable	Appropriate exposure or condition measure	Exposure measured similarly for cases and controls/both groups	Confounders addressed	Appropriate outcome measures	Appropriate exposure or follow‐up period	Appropriate statistical analysis	Follow‐up complete	Cases and controls appropriately matched	Same criteria to ID cases and controls	Groups free of exposure at study initiation	Inclusion criteria identified	Sample and setting described	Clear cause and effect	Compared participants received similar treatment	Control group	Multiple measures pre‐post intervention	Outcomes measured similarly across participants
Bernard et al. (2015)	Cross‐sectional	N/A	Y	Y	Y	Y	N/A	Y	N/A	N/A	N/A	N/A	Y	Y	N/A	N/A	N/A	N/A	N/A
Bryson et al. (2019)	Cohort	N/A	Y	N/A	Y	Y	Y	Y	Y	N/A	N/A	N/A	N/A	N/A	N/A	N/A	N/A	N/A	N/A
Bush et al. (2011)	Cohort	N/A	Y	N/A	Y	Y	Y	Y	Y	N/A	N/A	?	N/A	N/A	N/A	N/A	N/A	N/A	N/A
Chernego et al. (2019)	Case–control	?	Y	Y	Y	Y	Y	Y	N/A	Y	Y	N/A	N/A	N/A	N/A	N/A	N/A	N/A	N/A
Chryssanthopoulou et al. (2005)	Cross‐sectional	N/A	Y	?	Y	?	N/A	Y	N/A	N/A	N/A	N/A	Y	Y	N/A	N/A	N/A	N/A	N/A
Clowtis et al. (2016)	Cross‐sectional	N/A	Y	Y	Y	Y	N/A	Y	N/A	N/A	N/A	N/A	Y	Y	N/A	N/A	N/A	N/A	N/A
Dougherty et al. (2009)	Cross‐sectional	N/A	Y	Y	Y	Y	N/A	Y	N/A	N/A	N/A	N/A	Y	Y	N/A	N/A	N/A	N/A	N/A
Dougherty et al. (2011)	Quasi‐experimental	N/A	N/A	N/A	N/A	Y	N/A	Y	Y	N/A	N/A	N/A	N/A	N/A	Y	N/A	N	Y	N/A
Dougherty et al. (2013)	Quasi‐experimental	N/A	N/A	N/A	N/A	Y	N/A	Y	Y	N/A	N/A	N/A	N/A	N/A	Y	N/A	N	Y	N/A
Feldman et al. (2013)	Case–control	Y	N/A	N/A	N/A	N	N/A	Y	Y	Y	Y	N/A	N/A	N/A	N/A	N/A	N/A	N/A	N/A
Fries et al. (2008)	Quasi‐experimental	Y	N/A	N/A	N/A	Y	N/A	Y	Y	N/A	N/A	N/A	N/A	N/A	Y	Y	Y	Y	Y
Giuliano et al. (2018)	Quasi‐experimental	Y	N/A	N/A	N/A	Y	N/A	Y	Y	N/A	N/A	N/A	N/A	N/A	Y	Y	N	Y	Y
Kopala‐Sibley et al. (2017)	Quasi‐experimental	N/A	N/A	N/A	N/A	Y	N/A	Y	Y	N/A	N/A	N/A	N/A	N/A	Y	N/A	N	Y	N/A
Koss et al. (2014)	Case–control	Y	Y	Y	Y	Y	Y	Y	N/A	Y	Y	N/A	N/A	N/A	N/A	N/A	N/A	N/A	N/A
Koss et al. (2016)	Quasi‐experimental	Y	N/A	N/A	N/A	Y	N/A	Y	Y	N/A	N/A	N/A	N/A	N/A	Y	Y	N	Y	Y
Kroupina et al. (2012)	Cohort	N/A	Y	N/A	N	Y	Y	Y	Y	N/A	N/A	N/A	N/A	N/A	N/A	N/A	N/A	N/A	N/A
Laurent et al. (2014)	Cohort	N/A	Y	N/A	Y	Y	Y	Y	Y	N/A	N/A	N/A	N/A	N/A	N/A	N/A	N/A	N/A	N/A
Liu et al. (2020)	Cross‐sectional	N/A	Y	Y	Y	Y	N/A	Y	N/A	N/A	N/A	N/A	Y	Y	N/A	N/A	N/A	N/A	N/A
Ludmer et al. (2015)	Quasi‐experimental	Y	N/A	N/A	N/A	Y	N/A	N	Y	N/A	N/A	N/A	N/A	N/A	Y	Y	N	Y	Y
Lunkenheimer et al. (2018)	Quasi‐experimental	?	N/A	N/A	N/A	Y	N/A	Y	?	N/A	N/A	N/A	N/A	N/A	Y	Y	Y	Y	Y
Molenaar et al. (2019)	Cohort	N/A	Y	N/A	Y	Y	Y	Y	Y	N/A	N/A	N/A	N/A	N/A	N/A	N/A	N/A	N/A	N/A
Palmer et al. (2013)	Cohort	N/A	Y	N/A	N	Y	Y	Y	N	N/A	N/A	N/A	N/A	N/A	N/A	N/A	N/A	N/A	N/A
Paret et al. (2015)	Quasi‐experimental	Y	N/A	N/A	N/A	Y	N/A	Y	Y	N/A	N/A	N/A	N/A	N/A	Y	Y	N	Y	?
Saridjan et al. (2010)	Cross‐sectional	N/A	Y	Y	Y	Y	N/A	Y	N/A	N/A	N/A	N/A	Y	Y	N/A	N/A	N/A	N/A	N/A
Send, Bardtke, Gilles, Wolf, Sütterlin, Kirschbaum, et al. (2019)	Cohort	N/A	N/A	N/A	N/A	Y	N/A	Y	?	N/A	N/A	N	N/A	N/A	N/A	N/A	N/A	N/A	N/A
Send, Bardtke, Gilles, Wolf, Sütterlin, Wudy, et al. (2019)	Cohort	N/A	Y	N/A	Y	Y	Y	Y	Y	N/A	N/A	N/A	N/A	N/A	N/A	N/A	N/A	N/A	N/A
Skowron et al. (2014)	Quasi‐experimental	Y	N/A	N/A	N/A	Y	N/A	Y	N	N/A	N/A	N/A	N/A	N/A	Y	Y	N	Y	Y
Taylor et al. (2013)	Cohort	N/A	Y	N/A	Y	Y	Y	Y	Y	N/A	N/A	N/A	N/A	N/A	N/A	N/A	N/A	N/A	N/A
Vaghri et al. (2013)	Cross‐sectional	N/A	Y	Y	Y	Y	N/A	Y	N/A	N/A	N/A	N/A	Y	Y	N/A	N/A	N/A	N/A	N/A
von Klitzing et al. (2012)	Cross‐sectional	N/A	N/A	N/A	N/A	Y	N/A	Y	N	N/A	N/A	N/A	N	Y	N/A	N/A	N/A	N/A	N/A
Zalewski et al. (2012)	Cross‐sectional	N/A	Y	Y	Y	Y	N/A	Y	N/A	N/A	N/A	N/A	Y	Y	N/A	N/A	N/A	N/A	N/A
Zalewski et al. (2016)	Cohort	N/A	Y	N/A	Y	Y	Y	Y	Y	N/A	N/A	N/A	N/A	N/A	N/A	N/A	N/A	N/A	N/A

*Note*: Y = yes; N = no; ? = unclear; N/A = not applicable. Appraisal criteria listed under the “All studies” column had common evaluation elements across all study types from the Joanna Briggs Institute criteria. The remainder of the appraisal criteria was specific to study types.

**TABLE 2:** Summary of included studies (*n* = 32) organized by stress response measure then alphabetically

Study 1. Authors (year) 2. Type of study	Study objective	Child characteristics 1. *N* 2. Age 3. Race/ethnicity 4. Sex 5. SES	Psychosocial stressor	Stress response	Findings
Hair cortisol and/or cortisone					
1. Bryson et al. (2019) 2. Cohort	Determine associations between children's pre‐ and postnatal exposure to adversity at 2 years of age and hair cortisol at 2 years of age	1. *N* = 319 2. Age: *M* = 24.2 months (*SD* = 0.9) 3. Australian 4. 42% male 5. Mixed SES	Dichotomous risks 1. Young age at pregnancy (<23 years) versus not (≥23 years) 2. Maternal education (completed high school vs. not) 3. Marital status (living with partner or married vs. else) 4. Financial hardship 5. Paid employment versus not 6. “Drug problem” 7. “Alcohol problem” 8. Mother smokes during pregnancy 9. Housing problem 10. Family violence 11. Live in safe place Depression, anxiety, stress symptoms	Hair cortisol	• Higher hair cortisol associated with housing tenure of public rental, paying board, or living rent free; not living in a safe place; higher maternal stress symptoms (including intermittent and persistent vs. never) • Lower hair cortisol associated with housing problems versus not • Other associations not significant
1. Liu et al. (2020) 2. Cross‐sectional	Examine associations between caregiver depressive symptoms, caregiver social support, child effortful control, and child hair cortisol in a sample of a high‐risk, low‐income preschool‐aged children	1. *N* = 154 2. 2–5 years 3. Brazilian (primarily White or Mixed) 4. “slightly more girls than boys” *n* not provided 5. Poor, urban	Parent social support (Medical Outcomes Study Social Support Survey); maternal depression (Edinburgh Postnatal Depression Scale)	Hair cortisol	• Higher caregiver depression symptoms predicted higher child hair cortisol. Quadratic effects not significant. • Caregiver social support not associated with hair cortisol. • No hair cortisol differences based on caregiver type (e.g., father, mother, grandparent), parent relationship status, education level, child race, or sex.
1. Molenaar et al. (2019) 2. Cohort	Examine the relation of prenatal maternal symptoms of psychopathology and stress with offspring HPA axis activity at 6 years as measured by hair cortisol and cortisone concentrations.	1. *N* = 2456 2. Prenatal IVs and DV of hair cortisol and cortisone at 6 years 3. 50% Dutch 4. 47% male 5. SES‐diverse	Questionnaires on prenatal maternal symptoms of psychopathology (second trimester: Brief Symptom Inventory) and stress (second and third trimesters: Social Readjusting Rating Scale, Long Lasting Difficulties Questionnaire, Family Assessment Device, Pregnancy Outcome Questionnaire)	Hair cortisol and cortisone	• No significant associations between maternal psychopathology or maternal stress with child hair cortisol; however, trends emerged after adjusting for potential confounders (e.g., sociodemographics). • Higher child hair cortisone with overall prenatal maternal psychopathology, intrauterine stress, and individual symptoms of maternal psychopathology (interpersonal sensitivity, paranoid ideation, obsessive–compulsive symptoms, psychoticism). • After stratification by sex, results varied. In boys, increased maternal psychopathology was associated with higher child hair cortisone. In girls, increased maternal stress was associated with higher child hair cortisone.
1. Palmer et al. (2013) 2. Cohort	Determine sociodemographic and maternal and child factors associated with early childhood stress and socioemotional problems.	1. *N* = 297 2. 1‐year‐olds 3. Mostly Black or White in Tennessee, USA 4. Not provided 5. Full sample SES‐diverse, but not provided for hair cortisol	Maternal stress (Parenting Stress Index Short Form), depression (Edinburgh Postnatal Depression Scale), psychological symptoms (Brief Symptom Inventory), and risk for child abuse (Child Abuse Potential Inventory)	Hair cortisol	• All children (combined White and Black) had higher hair cortisol if they were Black as compared to White, exposed to higher parenting stress, lower maternal depression, and lower birth length. • Children who were Black had higher hair cortisol if their mothers had higher dysthymia, greater parenting stress, lower depression, and lower birth length. • Children who were White had higher hair cortisol if they had lower birth length at birth, greater length at 1 year, and higher parenting stress.
1. Vaghri et al. (2013) 2. Cross‐sectional	Examine relationship between SES and child HPA axis	1. *N* = 339 2. *M* = 4.6 years (*SD* = 0.5 years) 3. Majority White or Chinese in Vancouver, Canada 4. 49% boys 5. SES‐diverse	SES (parent education, income)	Hair cortisol	• Child lower hair cortisol related to higher mother and father education. Child hair cortisol not related to parent income.
Urinary cortisol and/or cortisone					
1. Fries et al. (2008) 2. Quasi‐experimental	Examine the effects of early neglect on the regulation of the HPA axis following social interactions	1. *N* = 39 2. M ∼ 54 months 3. Adopted from Russian and Romanian orphanages 4. Slightly less male than female 5. Mid‐high SES	Institutionalization versus not, severity of neglect in institution (parent report)	Urinary cortisol, (micrograms/dl creatinine) collected as basal samples and after experimental stressors. Calculated as basal levels and slopes after the stressor.	• Post‐institutionalized children with more severe neglect had higher basal and social reactivity cortisol levels.
1. Send, Bardtke, Gilles, Wolf, Sütterlin, Wudy, et al. (2019) 2. Cohort	Examine association between maternal prenatal stress and child nocturnal HPA axis regulation (cortisol, cortisone)	1. *N* = 280–371 (371 full sample) 2. 45 months (*SD* = 1 month) 3. German‐speaking and living in Germany 4. 46.6% male 5. SES not provided	Prenatal maternal perceived stress (Perceived Stress Scale), maternal diagnosed psychopathology (self‐report and Mini International Neuropsychiatric Interview), maternal anxiety (State‐Trait Anxiety Inventory), maternal depression (Edinburgh Postnatal Depression Scale)	Urine cortisol, cortisone, and cortisol/cortisone ratio. Collected as first morning urine.	• Children had lower nocturnal urine cortisol and cortisone with higher levels of maternal prenatal perceived stress, anxiety, and depression. • Children had lower nocturnal urine cortisone with higher expert‐rated psychopathology. • Null effects: child cortisol with maternal psychopathology (both expert and self‐rated); child ratio cortisone/(cortisone + cortisol) with all measures of maternal psychopathology, stress, anxiety, and depression
Diurnal salivary cortisol					
1. Bernard et al. (2015) 2. Cross‐sectional	Determine if poverty and involvement with child protective services had an effect on externalizing behavior through blunted diurnal cortisol patterns	1. *N* = 94 2. *M* = 4.93 years (*SD* = .58) 3. Diverse USA children 4. Sex: child protective status‐referred 64.2% male, 35.8% female; comparison 41.5% male, 58.5% female 5. SES: child protective status‐referred mean household income $15,645, comparison mean household income $53,489	Child abuse or neglect risk (child protective services referral); poverty status (income to needs ratio)	Diurnal salivary cortisol (upon waking and before bed across 3 days): included as individual measures in a structural equation model	• Children with risk of child abuse or neglect had more blunted cortisol slopes and lower wake‐up cortisol, no association with bedtime cortisol. • Children with higher levels of poverty had more blunted cortisol slopes, no association with wake‐up cortisol, and lower bedtime cortisol.
1. Bush et al. (2011) 2. Cohort	Determine the effects of cumulative contextual stressors on children's HPA axis regulation	1. *N* = 338 2. Age = *M* = 5.32 years (*SD* = 0.32) 3. Diverse USA children 4. 52% male 5. 75% of households having a member with at least college degree	Cumulative stressors 1. Ethnic minority status 2. Household income 3. Highest education level of any adult in household 4. Financial stress 5. Parenting overload 6. Marital conflict 7. Negative/anger expressiveness 8. Maternal depression Harsh and restrictive parenting	Diurnal salivary cortisol collected in fall and spring (twice daily: in the first and last 20 min of class, at the same time for three consecutive days). Calculated as AUCg	• Higher levels of cortisol were found for the following: ethnic minority status, adversity, and low SES (average of income and education) in both the fall and spring. • Some exceptions are the following: White children had higher cortisol at both high and low levels of SES. In the Spring, both higher and lower levels of adversity predicted lower levels of cortisol. Ethnic minority children had higher cortisol with lower adversity in the Fall and higher cortisol with higher adversity in the Spring. In the Fall, children had higher cortisol at both higher and lower levels of SES as compared to the mean.
1. Chernego et al. (2019) 2. Case–control	Assess cortisol differences between children institutionally and family reared	1. *N* = 73 2. ∼ 20–24 months of age 3. Children from and living in Russia 4. 56% Male 5. Mid‐slightly higher SES in St. Petersburg, Russia	12. Reared in institution versus in family	Diurnal salivary cortisol collected twice daily (within 30 min of waking and immediately before bed) during a 3‐day period and ∼4 months later. Calculated as intercepts and slopes across the day.	• Children reared in institutions had more blunted change in cortisol and had higher bedtime cortisol as compared to children reared in families. • Wake‐up cortisol did not differ between family‐ and institution‐reared children.
1. Chryssanthopoulou et al. (2005) 2. Cross‐sectional	Determine HPA axis functioning (salivary cortisol) in relation to children's environments (family, maternal occupation, childcare experiences)	1. *N* = 56 2. *M* = 47.4 months (*SD* = 6.5) 3. Mostly White in UK 4. 48.2% male 5. SES diverse	Sociodemographics (maternal age, educational attainment, marital status, and occupational status, family SES); childcare experiences (e.g., daycare); family environment and relations; maternal employment qualities, burnout, and full‐time status	Diurnal salivary cortisol collected immediately after awakening and the second in the early evening between 1700 and 1800. Repeated across 2 days. Calculated as mean awakening, evening, and total cortisol levels; and rate of change in cortisol from morning to evening.	• Children had higher evening and total cortisol if their mothers had low job role quality (compared to those with mothers with high job role quality). • Children had lower awakening cortisol if they had mothers with moderate levels of emotional exhaustion (compared to mothers with high emotional exhaustion). • Children had higher awakening cortisol if they attended less frequent childcare and had mothers with *higher emotional exhaustion*. • No associations between cortisol and maternal employment.
1. Clowtis et al. (2016) 2. Cross‐sectional	Examine the effects of maternal stress and interaction quality on maternal and child biological responses (salivary cortisol and testosterone) and child health in low‐SES setting	1. *N* = 50 2. *M* = 4.3 years 3. Primarily Hispanic in Texas, USA 4. Sex: 63.8% male, 36.2% female 5. Low SES	Maternal stress (Perceived Stress Scale) and depression (Center for Epidemiologic Studies Depression Scale), maternal–child engagement quality (free play measured with the Keys to Interactive Parenting Scale)	Diurnal salivary cortisol collected at “night” and in “morning.” Calculated as individual continuous variables.	• Children had lower morning cortisol if they had higher quality maternal–child engagement. No relations with child evening cortisol and maternal–child engagement. • Mother and child evening salivary cortisol strongly associated.
1. Dougherty et al. (2009) 2. Cross‐sectional	Examine relations among cortisol and maternal depression, parenting hostility, parent life stress, and child temperament.	1. *N* = 94 2. Age: *M* = 43.42 months (*SD* = 2.52) 3. Mostly White in the USA 4. Sex: 56.4% male 5. Mixed SES	Maternal depression and melancholia (Structured Clinical Interview for DSM‐IV), life stress (life events counted by the Preschool‐Age Psychiatric Assessment); observed parenting hostility (Teaching Task battery)	Diurnal salivary cortisol collected 30 min after wakening and 30 min before bedtime. Calculated as individual continuous variables.	• Children had significantly higher morning cortisol levels if they had mothers with melancholic depression mothers compared to mothers with no lifetime depression; no significant differences from mothers with nonmelancholic depression.
1. Koss et al. (2014) 2. Case–control	Examine changes in diurnal cortisol during the transition to family care in the first 2 years postadoption	1. *N* = 155 2. 26.4–32.3 months at initial study visit through 25 months later 3. Adopted overseas (diverse races) and mostly nonadopted White comparison group in the USA 4. About 50% male 5. Primarily high SES	Qualities of overseas institutionalized and foster care (parent interviews)	Diurnal salivary cortisol collected roughly 2, 9, 17, and 25 months after adoption. Calculated as latent variates and slopes in a structural equation model.	• Children post‐institutionalized and adopted from foster care overseas had blunted diurnal declines in cortisol compared to nonadopted children. • For children post‐institutionalized, better preadoptive social care predicted higher morning cortisol and steeper declines in diurnal cortisol. • Morning cortisol did not differ among the groups. • Morning or diurnal cortisol did not change over 2 years for the post‐institutionalized group.
1. Kroupina et al. (2012) 2. Cohort	Investigate 1) whether improvements in the social and emotional environment are associated with changes in HPA axis function and 2) explore whether HPA alterations related to early social adversity were associated with more compromised general development and social and emotional functioning post adoption.	1. N = 76 2. Mean:17 months (SD = 5) 3. Adopted from Eastern European orphanages, recruited in the USA 4. 49% male 5. SES not provided	Preadoption deprivation (age at adoption and physical growth at adoption)	Diurnal cortisol collected at 30 minutes after waking and 30 minutes before bedtime on two typical days at baseline and follow‐up. Calculated as average measures of wake‐up and bedtime and change scores (bedtime raw values – wake‐up raw values).	• Neither age at adoption nor growth average predicted baseline wake‐up cortisol. • Children older at adoption had lower bedtime cortisol levels. • Children with lower growth at adoption had higher wake‐up cortisol levels about 7 months after adoption.
1. Laurent et al. (2014) 2. Cohort	Examine early calibration of stress systems by testing links between adversity exposures, developmental stability of HPA axis activity, and behavior problems in a sample of adopted children.	1. *N* = 200 2. Assessments at 9, 18, and 27 months and 4.5 and 6 years 3. Children mixed races adopted and living in the USA 4. 57% male 5. Mostly white, middle‐class families	Cumulative adversity of parent: 1. Depression (Beck depression inventory) 2. Anxiety (Beck Anxiety Inventory, Spielberger State Trait Anxiety Inventory) 3. Negative life events checklist from 4. Social support survey 5. Marital instability (Marital Instability Index) 6. Financial need survey 7. Home chaos (Confusion, Hubbub, and Order Scale)	Diurnal cortisol collected as morning (30 min after awakening) and evening (when child put to bed) samples across three consecutive days at 4.5 and 6 years old. Calculated as individual values in a structural equation model.	• Mean and variable adversity from 9 months to 4.5 years not significantly associated with child cortisol. • Higher adversity predicted lower evening cortisol and less stability over time. • Increasing adversity from 4.5 to 6 years predicted higher morning cortisol at age 6. • Parent symptoms of depression and anxiety, negative life events, and marital instability at age 6 (controlling for 9 months) predicted lower and less stable child evening cortisol. • Increasing home chaos from 4.5 to 6 predicted more stable and slightly higher child morning cortisol.
1. Saridjan et al. (2010) 2. Cross‐sectional	How social disadvantage and family adversity are associated with HPA axis activity in early life	1. *N* = 366 2. 12–20 months 3. Dutch 4. 56.6% male 5. SES diverse	Young maternal age, single parenthood, low maternal education, low family income, maternal smoking during pregnancy, distress and hostility during pregnancy (Brief Symptom Inventory), poor family functioning during pregnancy (General Functioning subscale of the Family Assessment Device), low birth weight, postnatal parenting stress at 18 months (Dutch version of the Parenting Stress Index‐Short Form)	Diurnal salivary cortisol collected as five samples: immediately after awakening, 30 min later, around noon, between 3:00 p.m. and 4:00 p.m., and at bedtime. Calculated as AUC, diurnal cortisol slope, and cortisol awakening response.	• Underpowered to determine effects by each adversity determinant (i.e., young maternal age, single parenthood, low birth weight). • Infants had higher AUC level (diurnal) with low family income (compared to high family income), mothers experiencing parenting stress (compared to those with little or no parenting stress). • Infants had positive CAR if they had a low‐income family (compared to high income family), mothers smoked during pregnancy (CAR decline in those who did not smoke during pregnancy), birth weight above 4000 g (as compared to those between 3000 and 4000 g). • Null effects: Maternal age not associated with AUC, slope, or CAR. Infant sex not associated with differences in AUC, diurnal slope, or CAR. Infants of mothers with and without distress during pregnancy had no differences in AUC, slope, or CAR. Trend found in difference in diurnal slopes and CAR for infants of mothers with hostile behavior during pregnancy compared to infants of nonhostile mothers.
1. Zalewski et al. (2012) 2. Cross‐sectional	Examine the relation of low income and poverty to cortisol levels, and test potential pathways from low income to disruptions in cortisol through cumulative family risk and parenting.	1. *N* = 306 2. *M* = 37 months (*SD* = 0.84 months) 3. 64% European American 4. 50% boys 5. Mixed SES	Sociodemographic cumulative risk: parent income, education, marital status, age, depression (Center for Epidemiological Studies‐Depression), negative life events (General Life Events Schedule for Children), residential instability, household density), observed parent–child interaction (restricted play, free play, instructional activity, cleanup)	Diurnal salivary cortisol collected as 30 min after awakening and 30 min prior to bedtime for three consecutive days. Calculated as a diurnal slope value (subtract average evening from average morning), average morning, and average evening values.	• Children had lower morning cortisol associate with lower income, but this was not significant after controlling for cumulative risk (which predicted lower morning cortisol) • Children had low diurnal slope (i.e., blunted slope) if they had associations with having adolescent parent or had a single parent. • Children had lower morning levels and a low diurnal slope (i.e., blunted) associated with higher sociodemographic cumulative risk and higher maternal negativity. • Children had higher morning cortisol with higher maternal warmth (controlling for income and cumulative risk). • Children had steeper diurnal slope with more maternal responsiveness.
1. Zalewski et al. (2016) 2. Cohort	Examine profiles of HPA axis functioning and its relation to income and cumulative risk	1. *N* = 306 2. *M* = 37 months (*SD* = 0.84 months at time 1), then up to 63–67 months 3. 64% European American 4. 50% boys 5. Mixed SES	Repeated measures of income and cumulative risk: parent income, education, age, maternal depression (Center for Epidemiological Studies‐Depression Scale), negative life events (General Life Events Schedule for Children), residential instability (Changing households three or more times in previous 3 years), household density, family functioning (divorce during the family's lifetime)	Diurnal salivary cortisol collected as 30 min after awakening and 30 min prior to bedtime for 3 consecutive days. Calculated as a diurnal slope value (subtract average evening from average morning), average morning, and average evening values.	• Lower income was related to lower morning cortisol only at Time 4 and flatter diurnal slope at Times 3 and 4 • Higher cumulative risk was significantly related to lower morning cortisol levels at Times 1, 3, and 4 and with flatter diurnal slope levels at Times 3 and 4. • Both high and low income predicted profile membership in the consistently lower morning cortisol profile. • Average income predicted consistently average morning cortisol levels over time • Both high and low income and cumulative risk predicted this profile membership diurnal Profile 1 (flat slope, PM > AM levels across time) • Average income was related to diurnal slopes that increased in steepness.
Diurnal and reactive salivary cortisol					
1. Koss et al. (2016) 2. Quasi‐experimental	Examine longitudinal changes in children's stress reactivity after adoption	1. *N* = 167 2. 18–36 months at recruitment. Lab sessions through 24 months later 3. Multiracial children adopted overseas now in the USA 4. About 50% male 5. Primarily high SES	Laboratory stressors (variety of tasks) by adoption status (post‐institutionalized [PI], nonadopted [NA], adopted from foster care overseas [PFC])	Diurnal and reactive salivary cortisol. Calculated as estimate of the diurnal cortisol slope, one estimate of the diurnal cortisol intercept, one estimate of the laboratory reactivity slope, and one estimate of the laboratory reactivity intercept.	• Cortisol reactivity slopes did not differ between PFC‐NA and PFC‐PI. • PI and PFC children had blunted cortisol reactivity as compared to NA children. Nonadopted children had decreasing slopes over time as compared to the blunted slopes of PI children. • Children with longer early life adversity had greater degree of hypocortisolism factor and higher cortisol at the beginning of laboratory challenges. • No difference in basal cortisol by group. No race or ethnic differences in cortisol within groups.
Reactive salivary cortisol					
1. Dougherty et al. (2011) 2. Quasi‐experimental	Test the hypothesis that parenting behavior moderates the relation between parent depression history and child's stress reactivity (cortisol responses to a psychosocial stressor)	1. *N* = 160 2. Age: *M* = 43.5 months (*SD* = 2.8) 3. Mostly White in the suburban USA 4. Sex: 80 females 5. Mostly middle class	Parent depression disorder (Structured Clinical Interview for DSM‐IV) and observed parent hostility (Teaching Tasks battery)	Reactive salivary cortisol at baseline, then sequentially during and after lab stressors. Calculated as individual levels at baseline (intercept) and change in response to the stressor.	• Children had high and increasing cortisol levels only if they had parents with a history of depression during their first few years of life and hostility toward their child.
1. Dougherty et al. (2013) 2. Quasi‐experimental	Test the hypothesis that interactive effects of exposure to parental depression during early childhood and parental hostility affect development of young children's stress physiology and early emerging behavior problems	1. *N* = 146 2. Age: *M* = 49.9 months (*SD* = 9.8) 3. Race/Ethnicity: Mixed in Washington, DC, USA 4. Sex: About 49% male 5. SES: Mixed	Parent major depression disorder or depression disorder (Structured Clinical Interview for DMS‐IV); observed parent hostility (Teaching Tasks battery)	Reactive salivary cortisol at baseline, then sequentially during and after lab stressors. Calculated as mean values at five different time points and mean AUCi.	• Children had higher cortisol levels in response to a stressor if they were exposed to maternal depression during early childhood and if their parent showed high hostile parenting.
1. Kopala‐Sibley et al. (2017) 2. Quasi‐experimental	Examine whether HPA axis reactivity moderates the effect of the quality of the parent–child relationship on changes in temperament in early childhood	1. *N* = 126 2. 3 years, then 6 years 3. 96% White in the USA 4. 50.8% male 5. Most middle class	Observed parent–child interactions (Teaching Tasks Battery)	Reactive salivary cortisol (during Lab‐TAB) calculated as AUCg and AUCi	• Trend toward lower quality parent child relationship associated with greater increases in cortisol at age 3, no relationship with total cortisol
1. Ludmer et al. (2015) 2. Quasi‐experimental	Investigate the interaction effects of maternal depression and Infant stress genotypes (DRD2 and SLC6A3) on infant cortisol reactivity	1. *N* = 314 2. 16–17 months 3. Canadian: ∼75% White, 8.5% Asian 4. 52% male 5. High SES; most with college degree	Laboratory challenges (toy frustration procedure, strange situation procedure) Maternal depression (Beck Depression Inventory), observed parenting quality (Maternal Behavior Q‐Sort), self‐reported ancestry, age, education, relationship status; family income	Reactive salivary cortisol, calculated as AUCi	• Salivary AUC not correlated with family income, number of hours in out of home care, maternal age, education, parent relationship status, extensive crying during stressors, and race • Higher maternal depression predicted blunted reactivity in infants with “at least one A1 allele of DRD2 or with the 10/10 genotype of SLC6A3.”
1. Send, Bardtke, Gilles, Wolf, Sütterlin, Kirschbaum, et al. (2019) 2. Quasi‐experimental	Evaluate whether a stress test elicits a cortisol response in children; evaluate the relation between prenatal maternal stress and later child cortisol reactivity	1. *N* = 339 2. 45 months 3. German‐speaking and living in Germany 4. 44.2% male 5. SES not provided	Prenatal stress (Perceived Stress Scale), maternal psychopathology (self‐report and expert diagnosis with Mini International Neuropsychiatric Interview) with a stress paradigm for preschool children	Reactive salivary cortisol collected before the stress test, 10 min poststress test, 30 min poststress test, and 40 min poststress test. Calculated as individual measures in a general linear model for repeated measures.	• Children with lower cortisol levels after the stress test were those exposed to prenatal maternal psychopathology (both self‐report and expert rated) and those exposed to the highest quartile of maternal perceived stress (compared to the lower quartiles). • Null findings: child cortisol with maternal perceived stress (continuous measure).
1. von Klitzing et al. (2012) 2. Quasi‐experimental	Examine: (a) direct effects of family environment, peer victimization, and HPA axis dysregulation on kindergartener emotional symptoms; and (b) how interactions of these risk factors predict kindergartener emotional symptoms	1. *N* = 166 2. *M* = 5.23 years (*SD* = 0.35) 3. Majority German speaking, Swiss city (Basel) 4. 57.2% boys 5. Mainly middle class	Social–relational risk factors (emotional family environment [German version of the Family Environment Scales], peer victimization [Peer Victimization scales])	Reactive salivary cortisol collected at baseline and 30 min after a stressor. Calculated as individual variables and an increase calculation across the stressor.	• Child cortisol not directly correlated with any social–emotional risk factor. Interaction effects significant, but not focus of this study.
Reactive salivary cortisol and alpha‐amylase					
1. Feldman et al. (2013) 2. Quasi‐experimental	Examine stress biomarkers in children with or without PTSD from war exposure by maternal and child factors	1. *N* = 232 2. Age: 1.5–5 years 3. Race/Ethnicity: Israeli 4. Sex: 47.6% males 5. SES: mixed	± War exposure (location in Israel) and ± child PTSD (diagnostic Classification: Zero‐to‐Three), observed maternal–child interactions (Lab‐TAB), maternal PTSD (Posttraumatic Diagnostic Scale)	Reactive salivary cortisol and alpha‐amylase at baseline, then sequentially after lab stressors. Calculated as mean values at baseline, following the stressor, and at recovery.	• Children had consistently high cortisol and salivary alpha‐amylase if they were exposed to war but had no PTSD. • Children had increase in cortisol with stressors and decrease in cortisol at recovery (greater stress reactivity) and overall low salivary alpha‐amylase if they had no PTSD and no war exposure as compared to children exposed to war. • Children had consistently high cortisol and low cortisol reactivity if they were war exposed, whether or not they had PTSD. • Children had higher reactive sAA if they were war exposed but had no PTSD as compared to children with PTSD. • Mother and child cortisol and sAA were moderately correlated. • Children had lower cortisol if their mother had more PTSD symptoms. • Children had lower cortisol if they had higher reciprocity with their mother and lower maternal cortisol. • Children had higher sAA if they had higher reciprocity with their mother.
1. Taylor et al. (2013) 2. Cohort	Examine if sociodemographic risk, parenting, and temperament (effortful control) predicted child HPA axis and autonomic nervous system activity.	1. *N* = 148 2. 18, 30, and 72 months 3. >70% White in the USA, 15.7% Hispanic 4. 54.7% male 5. SES diverse	Sociodemographics at 18 months (education, age, number of children), intrusive–overcontrolling parenting (observed from free play, teaching paradigm, and cleanup task) at 30 months	Reactive salivary cortisol and alpha‐amylase at 72 months (but the task did not induce physiologic reactivity). Collected prior to the stressor task (not‐sharing task), 10 min after the task, and 20 min after the task. Calculated as individual variables in a structural equation model.	• Intrusive–overcontrolling parenting at 30 months predicted higher levels of children's cortisol and alpha‐amylase at 72 months. Higher demographic risk at 18 months had a trending direct relationship to predict lower cortisol levels at 72 months. • There was evidence of a mediation relationship in that higher demographic risk at 18 months predicted higher intrusive–overcontrolling parenting at 30 months, which then predicted higher cortisol and alpha‐amylase levels).
RSA					
1. Giuliano et al. (2018) 2. Quasi‐experimental	Examine whether children's ability to engage the parasympathetic nervous system impacts how risk affects inhibitory control	1. *N* = 184 2. 3–5 years (*M* = 3.81 years, *SD* = 0.76) 3. 80.1% White in the USA 4. 54.5% female 5. Mostly low SES	Unweighted cumulative risk 1. Low income < $30,000 2. Single parent 3. Maternal high school drop out 4. Family turmoil (upper quartile of Life Experiences Survey) 5. Child foster care experience 6. Exposure to violence (upper quartile of Children's Exposure to Community Violence Survey) 7. Child maltreatment factors (presence of maltreatment, severe maltreatment present)	RSA baseline and reactivity	• Cumulative risk not directly, significantly correlated with baseline RSA, task RSA, or RSA reactivity
1. Lunkenheimer et al. (2018) 2. Quasi‐experimental	Examine whether parent‐child coregulation of RSA differs by child maltreatment severity and subtype	1. N = 146 2. 3–5 years (M = 3.78 years, SD = 0.73 years) 3. > 90% White in USA 4. Sex not provided 5. Low‐middle SES	Parent‐child interactions (maternal scaffolding of using a toy, Duplo Puzzle task) by child maltreatment and subtype (Child Protective Services records)	Reactive RSA	• RSA did not differ by maltreatment status. • Lower child resting RSA associated with higher maternal education • Nonmaltreating dyads had RSA concordance. Maltreating dyads did not have RSA concordance, but this differed by abuse/neglect type. • Higher physical abuse predicted lower resting child RSA. Higher neglect severity predicted increasing child RSA.
1. Paret et al. (2015) 2. Quasi‐experimental	Determine how quality of the child–caregiver attachment was associated with the child's cardiac vagal reactivity	1. *N* = 48 2. *M* = 3 years, 9 months 3. Canadian 4. 48% male 5. Diverse SES	Child–caregiver attachment relationship (Preschool Attachment Classification System—see below) with lab stressor • Secure (comforted easily) • Insecure–avoidant (avoids mother and does not show significant emotion) • Insecure–ambivalent (follows mother around but whiny)	Reactive RSA conceptualized as cardiac vagal reactivity in response to novel social stressor	• During the stressor, secure children showed vagal withdrawal but ambivalent children did not show vagal withdrawal. • Heart rate and RSA did not change across stress episodes across the three types of attachment groups. • In response to the stressor, only the secure group had a meaningful decrease in RSA (reflecting vagal withdrawal) in contrast to the insecure–ambivalent group who did not have a physiologic response to the stressor (even controlling for baseline RSA).
1. Skowron et al. (2014) 2. Quasi‐experimental	Examine parasympathetic physiology as a moderator of the effects of early adversity (i.e., child abuse and neglect) on children's inhibitory control	1. *N* = 161 2. *M* = 3.71 years (*SD* = 0.72) 3. > 80% children White in the USA 4. 49.7% male 5. Mostly low SES	Effects of child maltreatment (Child Protective Services documentation coded by Maltreatment Classification System) during a laboratory stressor (Duplojoint, Waitjoint, Lab‐TAB)	Reactive RSA	• Child maltreatment status not directly associated with RSA at rest or during stressors. Moderations with inhibitory control significant, but were not the focus of this review.

*Note*: See original studies for original citations of standardized measures listed (e.g., the Center for Epidemiological Studies—Depression scale as used in Clowtis et al. [2016]).

Abbreviations: AUC, area under the curve; AUCg, area under the curve with respect to ground; AUCi, area under the curve with respect to the increase; CAR, cortisol awakening response; HPA, hypothalamus pituitary adrenal; *M*, mean; *n*, number; NA, nonadopted; PFC, adopted from foster care overseas; PI, post‐institutionalized; PTSD, posttraumatic stress disorder; RSA, respiratory sinus arrhythmia; sAA, salivary alpha amylase; *SD*, standard deviation; SES, socioeconomic status; UK, United Kingdom; USA, United States of America; vs, versus.

**TABLE 3:** Study characteristics (*n* = 32 studies)

Characteristic	*n*
Study demographics	
Unique cohorts	27
Approximate unique children	9107
Number of studies initiated by child age	
24 months	9
36 months	3
48 months	12
60 months	5
72 months	3
Study countries	
United States	17
Canada	3
Australia	1
Brazil	1
Russia or Eastern Europe	3
Western Europe	6
Israel	1
Number of studies that measured type of psychosocial stressor	
Caregiver resources	
Socioeconomic resources	13
Parent mental health	14
Child and family relationships	17
Physical environment	4
Overall family dynamics	5
Number of studies that measured type of stress response	
Hypothalamus pituitary adrenal axis	
Diurnal measures of salivary cortisol	12
Reactive salivary cortisol	10
Hair cortisol	5
Hair cortisone	1
Urinary cortisol	2
Urinary cortisone	1
Autonomic nervous system	
Respiratory sinus arrhythmia	4
Reactive salivary alpha amylase	2

*Note*: Number of studies will not add up to 32, as studies often measured more than one type of psychosocial stressor or stress response.

Across the 32 studies, there were 27 unique cohorts with at least 9107 unique children. The average sample size was 253 (range: 39–2546). Across the samples, the age range was 9–60 months old. Participants were from diverse socioeconomic backgrounds, sampled from primarily western nations: Australia, Canada, Germany, the Netherlands, Switzerland, the United Kingdom, the United States, Israel, Brazil, and Russia. Some samples included international adoptees who previously lived outside of a family context, such as in orphanages or similar institutions. Among all studies, there were over 100 unique findings on the relations between psychosocial stressors and stress responses. Most of the findings were measured as stress responses from the HPA axis. Only 16 unique findings, from six different studies, were based on the ANS (RSA and alpha‐amylase). An overview of the general direction of the stress response, quantified by psychosocial stressor, is in Table 4.

**TABLE 4:** Summary of direction of outcomes (*N* > 106) across studies

	Number of stress response outcomes		
Psychosocial stressor	Increased	Blunted	Null
Lower socioeconomic caregiver resources			
Lower family income	3^a^	2^a^	5
Lower parent education	3	0	3
Lower parent age	0	1	3
Minoritized family race/ethnicity	2^b^	0	3
Limited parent employment	1	0	2
Lower housing quality	2	1	0
Lower parent mental health resources			
Higher prenatal maternal depression	1	0	1
Higher postnatal maternal depression	5	2	1
Higher prenatal anxiety	1	2	4
Higher postnatal anxiety	4	2	0
Higher prenatal maternal psychopathology	2^b^	2	2
Higher caregiver social support	0	0	1
Higher parent substance use	1	0	1
Child and family relationships			
Decreased parent marital conditions	0	2	3
Risks of poorer family functioning	1	0	1
Increased child maltreatment	2	2	2
Poorer caregiver‐child interactions	5	4	3
Physical environment			
Nonbiological family contexts (institutionalized pre‐adoption)	2	6	6
Overall family dynamics			
Cumulative adversity	2	3^b^	2
Total	37^b^	27^b^	43

^a^ Highest and lowest income associated with curvilinear stress responses, either blunted or increased. See studies for more details.

^b^ Additional findings. See description of studies for more details.

With regard to overall study quality, we found that most studies had at least some bias (briefly listed in Table 1). We noted nine cross‐sectional studies, 10 cohort studies, two case‐control studies, and 11 quasi‐experimental studies. Most of the quasi‐experimental studies did not have a control group and did not conduct a power analysis, but the respective study authors usually noted that the study was exploratory in nature.

Below, the results are organized by the general type of ecobiodevelopmental psychosocial stressor: caregiver resources (organized by socioeconomic resources and parental mental health), child and family relationships, and physical environment. Some resulting studies measured cumulative stressors, which we categorize below as overall family dynamics. We discussed stress responses as output measures of the stress response systems along the HPA axis or ANS (e.g., salivary cortisol, RSA, etc.). We provided an overview of these findings by organizing results by direction of the stress response to the psychosocial stressor: increased, blunted, or null. In the presence of a psychosocial stressor, an increased stress response indicates that the stress response was heightened, blunted means that the stress response was dampened, and null indicates no significant stress response. We organized findings by direction of the general stress response due to substantial heterogeneity of findings and difficulty to combine stress measures due to different sampling strategies, laboratory techniques, and calculations. Finally, numbers of findings may not necessarily add up to the number of unique studies, as individual studies may have measured more than one type of stressor with more than one type of stress response.

### 3.2 Caregiver Resources and Child Stress Responses

#### 3.2.1 Socioeconomic Resources

Generally, there were 13 unique studies that examined relations between socioeconomic resources and child stress responses (Bernard et al., 2015; Bryson et al., 2019; Bush et al., 2011; Chryssanthopoulou et al., 2005; Koss et al., 2016; Liu et al., 2020; Ludmer et al., 2015; Lunkenheimer et al., 2018; Palmer et al., 2013; Saridjan et al., 2010; Vaghri et al., 2013; Zalewski et al., 2012, 2016). Most of the studies were appraised as including all suggested content by the Joanna Briggs Institute (Moola et al., 2020; Tufanaru et al., 2020) and thus were considered as having good methodological quality. Findings are discussed below in order of the socioeconomic resources measured: parent income, parent education, parent age, family race and ethnicity, parent employment, and housing type. Stress responses in the studies were measured as diurnal measures of salivary cortisol, reactive salivary cortisol, reactive salivary alpha‐amylase, and hair cortisol. Across all studies, findings were mixed.

##### Family Income

Eight unique studies measured associations between family income and child stress responses; five studies measured aspects of diurnal levels of salivary cortisol (Bernard et al., 2015; Bush et al., 2011; Saridjan et al., 2010; Zalewski et al., 2012, 2016), two explored relations with hair cortisol (Bryson et al., 2019; Vaghri et al., 2013), and one explored relations with reactive salivary cortisol (Ludmer et al., 2015). Relations of these studies were mixed, depending on how stress was measured. Overall, in response to living with lower family income, two studies found increased stress responses, one found blunted stress responses, two found opposite curvilinear responses, and five found nonsignificant (null) stress responses.

###### Increased Stress Responses

Two studies found lower levels of income were associated with higher diurnal levels of child cortisol. Bush et al. (2011) found that older diverse U.S. preschoolers had higher diurnal salivary cortisol with lower SES, a measure of averaged income and education. Similarly, Saridjan et al. (2010) found that young Dutch toddlers living with low family income had higher diurnal salivary cortisol and higher awakening cortisol compared to those with high family income.

###### Blunted Stress Responses

Bernard et al. (2015) found blunted diurnal cortisol slopes in older preschoolers living with higher levels of poverty.

###### Curvilinear Stress Responses

Zalewski et al. (2016) found several relationships, but notably found that young, mostly White U.S. preschoolers had blunted diurnal salivary cortisol slopes with lower and higher income as compared to average income (who had the steepest diurnal cortisol slopes). Yet, Bush et al. (2011) found that White children with either high or low SES (a composite of income and education) had higher diurnal salivary cortisol.

###### Null Stress Responses

Five studies found null relationships between income measures and point or average estimates of cortisol excretion. While earlier we reviewed that Bernard et al. (2015) found blunted diurnal cortisol slopes with higher levels of poverty, they did not find any specific diurnal point estimates of salivary cortisol (bedtime or wake up) associated with the child's poverty status. In another study with the same sample as Zalewski et al. (2016), Zalewski et al. (2012) found that preschoolers had lower morning salivary cortisol with lower income; however, this relationship was null after controlling for cumulative risks. Ludmer et al. (2015) did not find a significant correlation between reactive salivary cortisol area, measured as area under the curve (AUC), and family income. In the two studies that measured hair cortisol, Vaghri et al. (2013) found a null relationship between child hair cortisol and parent income in Canadian children; Bryson et al. (2019) found null relationships between hair cortisol and financial hardship in mixed‐SES Australian toddlers.

##### Parent Education

Six studies measured associations between parent education and child physiologic stress responses, measured with diurnal salivary cortisol (Bush et al., 2011), reactive salivary cortisol (Ludmer et al., 2015), reactive RSA (Lunkenheimer et al., 2018), or hair cortisol (Bryson et al., 2019; Liu et al., 2020; Vaghri et al., 2013). Relationships were mixed; three studies found that lower levels of parent education were associated with higher levels of stress responses and three studies found null relations.

###### Increased Stress Responses

As reviewed in the family income section, Bush et al. (2011) found that older preschoolers had higher diurnal salivary cortisol with lower SES (average of household income and highest level of household adult's education). Similarly, Lunkenheimer et al. (2018) found that preschoolers had higher (less variable) resting RSA with lower maternal education. Additionally, Vaghri et al. (2013) found that preschoolers had higher hair cortisol with lower parent education.

###### Null Stress Responses

Liu et al. (2020) and Bryson et al. (2019) found no significant relations between the hair cortisol of toddlers and preschoolers with their mothers’ or parents’ education level. Additionally, Ludmer et al. (2015) did not find any significant correlation between reactive salivary AUC and maternal education.

##### Parent Age

Four studies examined relations between maternal age and child stress responses with hair cortisol (Bryson et al., 2019), reactive salivary cortisol (Ludmer et al., 2015), and diurnal salivary cortisol (Saridjan et al., 2010; Zalewski et al., 2012). Of these studies, one found a blunted relationship and the other three found null relationships.

###### Blunted Stress Responses

Zalewski et al. (2012) found that young preschoolers had blunted diurnal salivary cortisol slopes if they had an adolescent parent compared to those who had adult‐age parents.

###### Null Stress Responses

Ludmer et al. (2015), Bryson et al. (2019), and Saridjan et al. (2010) found null relationships between maternal age and child stress responses. Specifically, Ludmer et al. (2015) showed a null relationship between maternal age and toddler reactive salivary cortisol levels. Bryson et al. (2019) showed a null relationship between maternal age and the hair cortisol in toddlers. Finally, Saridjan et al. (2010) also found a null relationship between maternal age and the salivary diurnal cortisol levels and awakening responses in young toddlers.

##### 3.2.1.1 Family Race and Ethnicity

Five unique studies examined relations between family race or ethnicity with child stress responses with hair cortisol (Liu et al., 2020; Palmer et al., 2013), reactive salivary cortisol (Ludmer et al., 2015), and diurnal salivary cortisol (Bush et al., 2011; Koss et al., 2016). Two studies found significantly higher stress hormones with minority status (Bush et al., 2011; Palmer et al., 2013), and three studies found null relations (Koss et al., 2016; Liu et al., 2020; Ludmer et al., 2015).

###### Increased stress responses

Palmer et al. (2013) found that U.S. toddlers had higher hair cortisol if they were Black as compared to White, with additional racial differences by maternal stress type and child growth. Bush et al. (2011) also found that older United States preschoolers had higher diurnal salivary cortisol if they were ethnically minoritized as compared to those who were White. Bush et al. (2011) also found several other racial and ethnic minority differences by an overall family adversity and SES, depending on the time of the school year.

###### Null stress responses

Liu et al. (2020) found that toddlers and preschoolers had no differences in hair cortisol by race. Similarly, Ludmer et al. (2015) found that toddlers had no significant changes in their reactive salivary cortisol by race. In a study on adoption status, including with institutionally reared children overseas, Koss et al. (2016) found that toddlers and preschoolers had no racial or ethnic differences in diurnal or reactive salivary cortisol by adoption group (institutionally reared overseas, foster care, and nonadopted).

##### Parent employment

Two unique studies that examined relations between parent employment and child stress responses measured hair cortisol (Bryson et al., 2019) and diurnal salivary cortisol (Chryssanthopoulou et al., 2005). One study found higher stress responses with lower parental job role quality, and two studies found null stress responses with parent employment status.

###### Increased stress responses

Chryssanthopoulou et al. (2005) showed that preschoolers had higher evening salivary cortisol and higher total diurnal salivary cortisol if their mother had low job role quality as compared to mothers who had high job role quality.

###### Null stress responses

Bryson et al. (2019) found no significant association between older Australian preschoolers’ hair cortisol and their parent's employment status (employed vs. not). Chryssanthopoulou et al. (2005) also did not find any relation between British preschooler's diurnal salivary cortisol level and their mother's employment status.

##### Housing

Only one unique study measured the relationship between characteristics of housing and child stress responses, measured with hair cortisol, and found both increased and blunted stress responses.

###### Increased stress responses

Bryson et al. (2019) found that Australian toddlers had marginally higher hair cortisol if they lived rent free, in a public rental, or “paying board” as compared to those who lived in fully owned, purchased, or private rental homes. The researchers also found that toddlers had higher hair cortisol if parents felt they were not “living in a safe place” (Bryson et al., 2019).

###### Blunted stress responses

Bryson et al. (2019) also found that those with “housing problems” had marginally lower hair cortisol compared to those without housing problems.

#### 3.2.2 Parental mental health resources

Generally, there were 14 unique studies that examined relations between parental mental health resources and child stress responses (Bryson et al., 2019; Chryssanthopoulou et al., 2005; Dougherty et al., 2009, 2011, 2013; Feldman et al., 2013; Laurent et al., 2014; Liu et al., 2020; Ludmer et al., 2015; Molenaar et al., 2019; Palmer et al., 2013; Saridjan et al., 2010; Send, Bardtke, Gilles, Wolf, Sütterlin, Kirschbaum, et al., 2019; Send, Bardtke, Gilles, Wolf, Sütterlin, Wudy, et al., 2019). The parent mental health resources examined were parental depression, stress, anxiety, psychopathology, and social support. The stress responses measured were diurnal and reactive salivary cortisol, hair cortisol, and urinary cortisol and cortisone. Across all studies, findings were mixed and directions are discussed below. Most of the studies were appraised as including all suggested content by the Joanna Briggs Institute (Moola et al., 2020; Tufanaru et al., 2020) and thus were appraised as having good methodological quality.

##### Prenatal maternal depression symptoms

One study measured relations between prenatal maternal depression symptoms and later child stress responses measured with urinary cortisol and cortisone (Send, Bardtke, Gilles, Wolf, Sütterlin, Wudy, et al., 2019). Findings were mixed. Send, Bardtke, Gilles, Wolf, Sütterlin, Wudy, et al. (2019) found that preschoolers had lower nocturnal urine cortisol and cortisone if their mother had higher prenatal depression. However, they did not find any relation between prenatal maternal depression and child nocturnal cortisone/(cortisone + cortisol) ratio at 45 months of age (Send, Bardtke, Gilles, Wolf, Sütterlin, Wudy, et al., 2019).

##### Postnatal maternal depression symptoms

Six studies examined relations between postnatal maternal depression and child stress responses, measured with diurnal salivary cortisol (Dougherty et al., 2009, 2011), reactive salivary cortisol (Dougherty et al., 2013; Ludmer et al., 2015), and hair cortisol (Liu et al., 2020; Palmer et al., 2013). Five studies found increased stress responses, two studies found blunted stress responses, and one study found null relations.

###### Increased stress responses

Dougherty et al. (2011, 2013) found that preschoolers had high diurnal salivary cortisol or high reactive salivary cortisol if their parent had a history of depression during the child's first few years of life and had hostility toward the child. Dougherty et al. (2009) found that preschoolers had higher morning diurnal salivary cortisol if their mother had melancholic depression compared to those with mothers who had no lifetime depression. Liu et al. (2020) found that toddlers and preschoolers had higher hair cortisol if their caregiver had higher depression. Palmer et al. (2013) showed that young toddlers had higher hair cortisol if they were Black and their mother had higher dysthymia.

###### Blunted stress responses

Ludmer et al. (2015) found that toddlers with at least one A1 allele of DRD2 or with the 10/10 genotype of SLC6A3 had blunted reactive salivary cortisol if their mother had higher depression. Palmer et al. (2013) found that hair cortisol was lower in children if the mother had higher depression.

###### Null stress responses

Dougherty et al. (2009) found no significant relation between preschooler diurnal salivary cortisol levels in mothers with nonmelancholic depression compared to other types of depression.

##### Prenatal maternal stress and anxiety

Four unique studies examined relations between prenatal maternal stress or anxiety and child stress responses, measured by hair cortisone (Molenaar et al., 2019), diurnal salivary cortisol (Saridjan et al., 2010), reactive salivary cortisol (Send, Bardtke, Gilles, Wolf, Sütterlin, Kirschbaum, et al., 2019), and urinary cortisol and cortisone (Send, Bardtke, Gilles, Wolf, Sütterlin, Wudy, et al., 2019). Findings were again mixed, with one study finding increased stress responses, two finding blunted stress responses, and four finding null responses.

###### Increased stress responses

Molenaar et al. (2019) found that 72‐month‐olds had higher hair cortisone if they experienced higher levels of intrauterine maternal stress.

###### Blunted stress responses

Send, Bardtke, Gilles, Wolf, Sütterlin, Kirschbaum, et al. (2019) found that preschoolers had lower post‐stressor reactive salivary cortisol if they were exposed to the highest quartile of prenatal maternal perceived stress (compared to lower quartiles). Send, Bardtke, Gilles, Wolf, Sütterlin, Wudy, et al. (2019) found that preschoolers had lower nocturnal urine cortisol and cortisone if they were exposed to higher levels of maternal prenatal perceived stress or higher anxiety.

###### Null stress responses

Molenaar et al. (2019) found no relations between the child's hair cortisol and general prenatal maternal stress. Similarly, Saridjan et al. (2010) found no relationship between toddlers’ diurnal salivary cortisol with prenatal maternal distress. Send, Bardtke, Gilles, Wolf, Sütterlin, Kirschbaum, et al. (2019) also found null relations between preschoolers’ reactive salivary cortisol and prenatal maternal perceived stress. Finally, Send, Bardtke, Gilles, Wolf, Sütterlin, Wudy, et al. (2019) found a null relation between preschoolers’ urinary cortisone/(cortisone + cortisol) ratio and prenatal maternal stress or anxiety.

##### Postnatal maternal stress and anxiety

Five studies examined relations between postnatal maternal stress or anxiety and child stress responses, measured by diurnal salivary cortisol (Chryssanthopoulou et al., 2005; Laurent et al., 2014), reactive salivary cortisol (Feldman et al., 2013), and hair cortisol (Bryson et al., 2019; Palmer et al., 2013). Findings were again mixed, with four studies finding increased stress responses and two finding blunted stress responses. Children typically had increased stress responses with greater maternal stress but blunted stress responses if their parent had clinical stress diagnoses.

###### Increased stress responses

Bryson et al. (2019) found that toddlers had higher hair cortisol if their mother had higher stress symptoms. Palmer et al. (2013) found that young toddlers had higher hair cortisol if they experienced greater levels of parenting stress. Chryssanthopoulou et al. (2005) found that preschoolers had higher awakening diurnal salivary cortisol if their mother had higher emotional exhaustion. Saridjan et al. (2010) found that young toddlers had higher diurnal salivary cortisol if their mothers had higher levels of parenting stress, as compared to little or no stress.

###### Blunted stress responses

Laurent et al. (2014) found that children had lower and less stable evening diurnal salivary cortisol with higher levels of parent anxiety at 6 years of age. Feldman et al. (2013) found that toddlers and preschoolers had lower reactive salivary cortisol if their mother had more posttraumatic stress disorder symptoms.

##### Prenatal maternal psychopathology

There were three unique studies that measured relations between prenatal maternal psychopathology and child stress responses, measured with hair cortisone and cortisol (Molenaar et al., 2019), reactive salivary cortisol (Send, Bardtke, Gilles, Wolf, Sütterlin, Kirschbaum, et al., 2019), and urine cortisol and urine cortisone (Send, Bardtke, Gilles, Wolf, Sütterlin, Wudy, et al., 2019). Findings were again mixed, with one study finding increased stress responses, two finding blunted stress responses, and two finding null responses.

###### Increased stress responses

Molenaar et al. (2019) found that 72‐month‐olds had higher hair cortisone if they were exposed to symptoms of prenatal maternal psychopathology during the second trimester. Yet, this relation differed by child sex and the type of prenatal maternal psychopathology (Molenaar et al., 2019).

###### Blunted stress responses

Send, Bardtke, Gilles, Wolf, Sütterlin, Kirschbaum, et al. (2019) found that preschoolers had lower post‐stressor reactive salivary cortisol if they were also exposed to prenatal maternal psychopathology (primarily measured in the third trimester). Similarly, Send, Bardtke, Gilles, Wolf, Sütterlin, Wudy, et al. (2019) found that preschoolers had lower nocturnal urine cortisone with higher expert‐rated prenatal maternal psychopathology.

###### Null stress responses

While Molenaar et al. (2019) found increased stress responses between prenatal maternal psychopathology and the 72‐month‐old's hair cortisone, they found no relationship with hair cortisol. Trends did emerge, however, after adjusting for potential confounders such as sociodemographics. Send, Bardtke, Gilles, Wolf, Sütterlin, Wudy, et al. (2019) found null relationships between maternal prenatal psychopathology and nocturnal urine cortisol and urine cortisol/cortisone ratio.

##### Caregiver social support

One study examined relations between caregiver social support and child stress responses, measured by hair cortisol. Liu et al. (2020) found no significant relations between toddlers’ and preschoolers’ hair cortisol and their caregiver's perceived social support.

##### Parent substance use

Two studies examined relations between parent drug or alcohol use and child stress responses, measured with hair cortisol and diurnal salivary cortisol. Findings were also mixed, with one finding increased stress responses and one finding a null response.

###### Increased stress responses

Saridjan et al. (2010) found that young toddlers had an increased diurnal salivary cortisol awakening response (cortisol increased from awakening to about 30 min later) if they were exposed to prenatal maternal smoking, but toddlers not exposed to prenatal maternal smoking had a negative diurnal salivary cortisol awakening response.

###### Null stress responses

Bryson et al. (2019) found null relations between toddler hair cortisol and parent alcohol, drug, or smoking “problems.”

### 3.3 Child and family relationships and child stress responses

There were 17 studies that examined relations between child and family relationships and child stress responses (Bernard et al., 2015; Bryson et al., 2019; Clowtis et al., 2016; Dougherty et al., 2011, 2013; Feldman et al., 2013; Fries et al., 2008; Kopala‐Sibley et al., 2017; Laurent et al., 2014; Liu et al., 2020; Ludmer et al., 2015; Lunkenheimer et al., 2018; Paret et al., 2015; Skowron et al., 2014; Taylor et al., 2013; von Klitzing et al., 2012; Zalewski et al., 2012). The child and family relationships examined were parent marital conditions, family functioning, child maltreatment, and child–caregiver interactions. The studies measured stress responses as reactive and diurnal salivary cortisol, hair cortisol, reactive salivary alpha‐amylase, RSA, and basal and reactive urinary cortisol. Across all studies, findings were mixed and details are outlined below. Most of the studies were appraised as including all suggested content by the Joanna Briggs Institute (Moola et al., 2020; Tufanaru et al., 2020) and thus were appraised as having good methodological quality. However, the Feldman et al. (2013) authors admitted to having inappropriate timing related to their measure of salivary alpha‐amylase. Further, many of the quasi‐experimental studies did not include control groups.

#### 3.3.1 Parent marital conditions

Five studies examined relations between parent marital conditions and child stress responses with reactive salivary cortisol (Ludmer et al., 2015), hair cortisol (Bryson et al., 2019; Liu et al., 2020), and diurnal salivary cortisol (Laurent et al., 2014; Zalewski et al., 2012). Findings showed two blunted relations and three null relations.

##### Blunted stress responses

Laurent et al. (2014) found that children had lower and less stable evening diurnal salivary cortisol if their parents had marital instability. Similarly, Zalewski et al. (2012) also found that young preschoolers had lower or blunted diurnal salivary cortisol slopes if they lived with a single parent as compared to married parents.

##### Null stress responses

Ludmer et al. (2015) found that toddlers had no differences in their reactive salivary cortisol levels by “parent relationship status.” Similarly, Bryson et al. (2019) and Liu et al. (2020) found no differences in toddler or preschooler hair cortisol levels by parent relationship status.

#### 3.3.2 Family functioning

Two studies examined relations between family functioning and child stress responses; one found increased stress responses and one found null responses. Laurent et al. (2014) found that children had higher child morning diurnal salivary cortisol with increasing home chaos from 4.5 to 6 years. However, von Klitzing et al. (2012) found that older preschoolers had no significant relations between family social–emotional risk factors and their reactive salivary cortisol.

#### 3.3.3 Child maltreatment in biological and nonbiological family contexts

Four studies examined relations between conditions of child maltreatment and child stress responses with urinary cortisol (Fries et al., 2008), RSA (Lunkenheimer et al., 2018; Skowron et al., 2014), and diurnal salivary cortisol (Bernard et al., 2015). Findings were mixed, with two finding increased stress responses, two finding blunted stress responses, and two finding null stress responses.

##### Increased stress responses

First, Fries et al. (2008) found that post‐institutionalized preschoolers had higher basal and reactive urinary cortisol if they experienced more severe neglect during their institutionalization. Additionally, Lunkenheimer et al. (2018) found increasing resting RSA (less variability) with higher neglect severity.

##### Blunted stress responses

Bernard et al. (2015) found that older preschoolers had blunted/lower wake‐up or diurnal salivary cortisol slopes if they experienced any child abuse or neglect as compared to not. Lunkenheimer et al. (2018) found that preschoolers had lower resting RSA with higher physical abuse.

##### Null stress responses

Bernard et al. (2015) found no significant relations between bedtime diurnal salivary cortisol and child abuse or neglect. Skowron et al. (2014) also found no relations between preschoolers’ maltreatment status and their resting or reactive RSA.

#### 3.3.4 Caregiver–child interactions

Eight studies examined relations between parent–child interactions and child stress responses measured with RSA (Paret et al., 2015), reactive salivary cortisol (Dougherty et al., 2013; Feldman et al., 2013; Kopala‐Sibley et al., 2017; Taylor et al., 2013), reactive salivary alpha‐amylase (Feldman et al., 2013; Taylor et al., 2013), and diurnal salivary cortisol (Clowtis et al., 2016; Dougherty et al., 2011; Zalewski et al., 2012). Generally, about 75% of studies found dysregulated stress responses. Five studies found increased stress responses, four found blunted, and three found null stress responses.

##### Increased stress responses

Feldman et al. (2013) found that toddlers and preschoolers had higher reactive salivary cortisol if they had lower reciprocity with their mother. Kopala‐Sibley et al. (2017) found that preschoolers had a higher increase in their reactive salivary cortisol if they had an observed lower quality parent–child relationship. Taylor et al. (2013) found that toddlers and preschoolers had higher reactive salivary cortisol at 72 months of age if they experienced intrusive–overcontrolling parenting at 30 months of age (predicted by higher demographic risks at 18 months of age). Dougherty et al. (2011) found that preschoolers had higher diurnal salivary cortisol if their parent had a history of depression during first few years of life and was hostile toward the child. Clowtis et al. (2016) found that preschoolers had higher morning diurnal salivary cortisol if they had lower quality maternal–child engagement.

##### Blunted stress responses

Zalewski et al. (2012) found that young preschoolers had blunted diurnal salivary cortisol slope and lower morning diurnal salivary cortisol with higher maternal negativity. Additionally, preschoolers had lower morning diurnal salivary cortisol with lower maternal warmth and blunted decline in diurnal salivary cortisol slopes with lower maternal responsiveness (Zalewski et al., 2012). Feldman et al. (2013) found that toddlers and preschoolers had lower reactive salivary alpha‐amylase if they had lower reciprocity with their mother.

Paret et al. (2015) examined preschoolers across three attachment types: secure, ambivalent, and insecure–ambivalent. Paret et al. (2015) found that preschoolers had a decrease in their reactive RSA (vagal withdrawal) if they had secure attachment with their parent, in contrast to those with an insecure–ambivalent group (who did not have a physiologic response to the laboratory stress paradigm).

##### Null stress responses

Paret et al. (2015) did not find any changes in RSA reactivity (vagal withdrawal) in children with ambivalent attachment. Bryson et al. (2019) found that there was a null relationship between toddler hair cortisol and the presence of family violence. Clowtis et al. (2016) found null relations between preschoolers’ evening diurnal salivary cortisol and quality of maternal–child engagement.

### 3.4 Physical environment and child stress responses

#### 3.4.1 Nonbiological family contexts (adoption and institutional rearing characteristics)

There were four studies that examined relations between the physical environment, primarily as nonbiological family contexts, and child stress responses. Four studies examined relations between characteristics of adoption and institutional rearing and child's stress responses, measured by diurnal salivary cortisol (Chernego et al., 2019; Koss et al., 2014; Kroupina et al., 2012) and reactive salivary cortisol (Koss et al., 2016). Results again were mixed, with two findings with increased stress responses, five findings with blunted stress responses, and six findings with null responses. Most of the studies were appraised as including all suggested content by the Joanna Briggs Institute (Moola et al., 2020; Tufanaru et al., 2020) and thus were appraised as having good methodological quality.

##### Increased stress responses

Chernego et al. (2019) found that toddlers had higher bedtime diurnal salivary cortisol if they were reared in institutions as compared to reared in families. Koss et al. (2016) found that the longer children lived in institutions and foster care, the higher their reactive salivary cortisol levels were at the beginning of a laboratory challenge.

##### Blunted stress responses

Koss et al. (2016) also found that toddlers and preschoolers post‐institutionalized and adopted from foster care overseas had blunted diurnal and reactive salivary cortisol compared to nonadopted children. Additionally, Koss et al. (2016) found that the longer children lived in institutions and foster care, the greater degree of hypocortisolism over the day.

In likely the same sample, Koss et al. (2014) found that toddlers and preschoolers post‐institutionalized and adopted from foster care overseas had blunted diurnal salivary cortisol compared to nonadopted children. The researchers also found that toddlers and preschoolers had lower morning diurnal salivary cortisol and more blunted declines in diurnal salivary cortisol if they had worse preadoptive social care after post‐institutionalization (Koss et al., 2014).

Chernego et al. (2019) also found that toddlers had blunted diurnal salivary cortisol if they were reared in institutions compared to families. Finally, Kroupina et al. (2012) found that toddlers had lower bedtime diurnal salivary cortisol the older they were at adoption.

##### Null stress responses

Chernego et al. (2019) did not find a significant relation between toddler awakening diurnal salivary cortisol and rearing by family or institution. Kroupina et al. (2012) found no significant relationship between toddler awakening diurnal salivary cortisol and age of adoption. Similarly, Koss et al. (2014, 2016) found that morning diurnal salivary cortisol did not differ among the foster care, institutionalized, and nonadopted groups of children reared in families. Additionally, Koss et al. (2016) did not find that cortisol reactivity slopes differed from those who were adopted from foster care overseas as compared to those nonadopted or those who were adopted after living in an institution. Koss et al. (2014) also did not find that the group of post‐institutionalized children had changes in their morning and diurnal salivary cortisol levels over 2 years.

### 3.5 Overall family dynamics and child stress responses

Overall, there were five studies that examined relations between overall family dynamics and child stress responses (Bush et al., 2011; Giuliano et al., 2018; Laurent et al., 2014; Zalewski et al., 2012, 2016). The studies measured stress responses as diurnal salivary cortisol and RSA. Across all studies, findings were again mixed, with three studies showing blunted stress responses and two studies finding null stress responses. Most of the studies were appraised as including all suggested content by the Joanna Briggs Institute (Moola et al., 2020; Tufanaru et al., 2020) and thus were appraised as having good methodological quality.

#### 3.5.1 Increased stress responses

Bush et al. (2011) found that with higher levels of cumulative adverse family dynamics (financial stress, parenting overload, marital conflict, family anger expression, maternal depression, and harsh parenting), older preschoolers had higher diurnal salivary cortisol. Laurent et al. (2014) showed that increasing levels of cumulative family adversity (parent depression, anxiety, negative life events, social support, marital instability, financial need, home chaos) from 4.5 to 6 years of age were associated with higher morning diurnal salivary cortisol at age 6.

#### 3.5.2 Blunted stress responses

Zalewski et al. examined the same group of preschoolers in two studies and found blunted stress responses to cumulative adverse family dynamics. First, Zalewski et al. (2012) found that with higher sociodemographic cumulative risks (parent income, education, marital status, age, depression, negative life events, residential instability, household density, observed parent–child interaction), young preschoolers had a blunted diurnal salivary cortisol slope and a lower morning diurnal salivary cortisol level. Zalewski et al. (2016) also found similar findings in a longitudinal study, in that higher cumulative risk (parent income, education, age, maternal depression and negative life events, residential instability, household density, family functioning) predicted that young preschoolers would have blunted diurnal salivary cortisol membership or lower morning diurnal salivary cortisol at several time points. Laurent et al. (2014) showed that increasing cumulative family adversity was associated with lower evening diurnal salivary cortisol and less stability.

#### 3.5.3 Null stress responses

Giuliano et al. (2018) found a null relationship between cumulative family adversity (dichotomous risks of low income <$30,000 yearly, single‐parent status, maternal high school dropout, upper quartile of family turmoil, child foster care experience, upper quartile of exposure to violence, child maltreatment) and preschooler RSA baseline or reactivity. Additionally, Laurent et al. (2014) found no relationship between diurnal salivary cortisol and mean and variable cumulative adversity from age 9 months to 4.5 years.

## 4 DISCUSSION

This is the first known systematic review on evidence regarding the associations between adverse psychosocial stressors at the family level, outlined by the ecobiodevelopmental model, and stress responses, along the HPA axis and ANS, in children 1–6 years of age. From 32 observational and quasi‐experimental studies, we examined over 100 findings from more than 9000 unique children in 27 different cohorts. Only 16 stress response findings were measured from the ANS; the remainder of the findings were from measuring stress responses along the HPA axis. Overall, the direction of the findings was roughly evenly mixed (see Table 4 for a review). Specifically, in response to a psychosocial stressor, approximately one third of overall findings resulted in increased stress responses, one third of findings resulted in blunted stress responses, and one third of findings resulted in nonsignificant stress responses. In other words, nearly two thirds of findings indicated an increased or blunted stress response in the child, or overall dysregulation to psychosocial stressors, and one third of findings were insignificant. Our quality appraisal of the studies did not yield any major patterns of untoward bias, except that most quasi‐experimental studies did not have a control group. Some reasons for the mixed findings may be related to characteristics of the child (i.e., moderators), characteristics of the stressor, how the stress response was measured, unmeasured variables (e.g., caregiving buffering), researcher degrees of freedom, or publication bias. Still, the mixed results align with early childhood ecobiodevelopmental theories outlining that child characteristics (e.g., the vulnerability of child to different stressors), stressor characteristics (e.g., type, duration, and severity), and buffering support to the child (e.g., parenting style) may shape child stress responses (Belsky, 2022; Gunnar & Quevedo, 2007; Shonkoff et al., 2012). Given that experiencing early childhood stress can advance skills or shape adverse outcomes later in life (McLaughlin et al., 2015; Shonkoff et al., 2012), these findings have important implications on the field of early childhood stress, related child outcomes, and potential targets for intervention. Below includes a discussion on the significance of dysregulated stress responses to theory‐based family‐level psychosocial stressors, notable findings, limitations, and future research.

In this study, we examined family‐level psychosocial stressors in accordance with ecobiodevelopmental theory (Shonkoff et al., 2012). Particularly in early childhood, the family environment—including stable and sufficient resources surrounding socioeconomics, mental health, and relationships—is critical to early childhood development. We anticipated that fewer resources and more unstable family environments or conditions of caregivers would be associated with more dysfunction of the child's stress response system, manifested as either heightened or dampened stress responses. We found nearly two thirds of the results suggested that children have either blunted or increased physiologic stress responses to psychosocial stressors, suggesting overall dysregulation of their stress response systems to psychosocial stressors. These mixed findings are similar to adolescent and adult research, where stressors have the capacity to increase or dampen stress responses (Ford et al., 2019; Miller et al., 2007). While stressors for children and adults differ, we note that children may be using similar physiologic stress response mechanisms to manage a perceptually significant stressor. That is, blunting responses to a stressor may represent withdrawal and disengagement from a stressor, which could represent an early developmental manifestation of “bracing oneself” when the child is not able to physically or psychologically “escape” from a difficult situation (Miller et al., 2007). Conversely, increased stress responses may represent the child's perception of being able to physiologically manage the stressor, represented by activating and mobilizing cellular and molecular resources to fight or flee the stressor (Miller et al., 2007). However, there is little consensus as to whether the blunted or increased stress response may be a positive, tolerable, or toxic stress response due to the complexity of the stress response system in humans and the multitude of social contexts and interactions (e.g., buffering from the love and care of an adult) that result in physiological stress responses that help a child manage their lived environment. Yet, emerging research suggests that blunted stress activity to psychosocial stressors is associated with poorer impulse control, which may represent one's difficulty navigating toward healthier life choices (Lovallo et al., 2019). Consequently, the combination of blunted stress activity and poor impulse control could represent a toxic maladjustment to stressors.

One of the reasons for the dysregulated stress responses could be related to the characteristics of the stressor. According to the ecobiodevelopmental model and other researchers, we know that a child's physiologic stress response can be influenced by the type, duration, and severity of the stressor (Gunnar & Quevedo, 2007; Miller et al., 2007; Shonkoff et al., 2012). In our study, the types of psychosocial stressors primarily studied were family resources (*n* = 27), followed by child–family relationships (*n* = 17) and the physical environment (*n* = 4). Of the family resources, 13 studies were on evaluating socioeconomic resources and 14 on parent mental health. Indicators of SES‐related family resources primarily included parent income, education, and race/ethnicity—factors that could also arguably be considered as of chronic duration.

With regard to parent income, we interestingly found that children might have dysregulated stress responses with very low or very high income. Yet, researchers found both increased and blunted stress responses. At lower and higher incomes, Bush et al. (2011) showed that White children had higher diurnal salivary cortisol but Zalewski et al. (2016) found blunted diurnal salivary cortisol slopes. The stressors that low‐ or high‐income individuals experience are likely vastly different, but there also may be some similarities. While individuals with low income likely are experiencing stress from not having enough resources to meet basic needs, individuals with high income may have added additional resources to their home that need to be managed, contributing to stress. Zalewski et al. (2016) theorize that children from both of these environments may be experiencing different types of “isolation” as compared to middle‐income families. For example, low‐income parents may often be away from their children or stressed due to having to work several jobs to make ends meet. Higher income parents may work long hours or be involved in commitments related to having and managing a higher income lifestyle and thus may also have to leave their children more often. However, it may also be that caregivers with more income are more likely to have the resources necessary to help buffer children from severe psychosocial stressors than caregivers living in poverty, and thus the nuances of the curvilinear stress responses may not capture the subtleties of the severity of extreme lived experiences. We may also not be able to ascertain the severity of the stressor, as this may depend on the (physiologically undetected perception of the child. More research will be needed to explore these curvilinear relationships and associated mechanisms.

Another common psychosocial stressor we found in the literature was parent mental health—specifically maternal mental health. Maternal mental health (depression: *n* = 7, stress and anxiety: *n* = 9) was assessed more frequently postnatally compared to prenatally. We found that children typically had increased physiologic stress responses to greater maternal stress symptoms (depression and anxiety) but blunted physiologic responses if the mother reported a clinical stress‐related diagnosis. Further, we found more consistent stress response dysregulation if the child experienced maternal depression or anxiety after childbirth (*n* = 7 relations with dysregulated stress responses and *n* = 1 relation with null findings) as compared to prenatally (*n* = 2 relations with dysregulated stress responses and *n* = 5 relations with null findings). Adding to a wide body of literature showing how maternal depression can affect child outcomes (Goodman & Gotlib, 1999; Jacques et al., 2019), we help delineate how it is related to child stress responses. Perhaps one mechanism for this, delineated by the ecobiodevelopmental model, is that the child's stress response systems become primed and regulated with early experiences (Shonkoff et al., 2012). For example, while the mother is exhibiting depression or anxiety symptoms, something about the mother's affect or specific interactions with the child may prime the child's stress responses to manage these interactions with the depressed or anxious mother. The child may “brace” themselves or activate molecular and cellular resources to manage the interaction with their anxious or depressed mother. This may be one mechanism for how early maternal depression and anxiety may shape how a child may manage interactions with other individuals throughout life. The lack of significant findings of prenatal anxiety and depression as compared to postnatal may indicate that the child's face‐to‐face interactions with the mother's affect may be particularly important for priming stress responses.

One final notable finding was that nearly 75% of findings showed a dysregulated stress response if a child encountered poorer interactions with their caregiver. Poor interactions with a caregiver were often with the mother, and included measures such as lower reciprocity, lower synchrony during observed laboratory tasks, intrusive–overcontrolling parenting, hostility, engagement, negativity, lower warmth, lower reciprocity, and an insecure–ambivalent attachment. The studies that found null responses measured ambivalent attachment, a history of family violence, and maternal–child engagement. These findings primarily suggest that experiencing negative interactions with a primary caregiver may prompt the stress response system to manage interaction, similarly to how a child may physiologically “learn” to manage interactions with a depressed or anxious mother. These findings also suggest that it may be difficult for the child to receive sufficient “buffering” from this primary caregiver from other types of psychosocial stressors. Hence, moving forward with research, it may be critical to examine caregiver–child interactions as both a stressor and as a potential moderator of a child's stress response to other psychosocial stressors, such as living with poverty.

We did not observe a consistent measure of a physiologic stress response in children from the reviewed studies. However, we observed more frequent assessments of the HPA axis (approximately 90 findings) compared to the ANS (approximately 16 findings). Most often, HPA assessments were diurnal measures of salivary cortisol, including area under the curve and slopes from morning to night, and measures of reactive salivary cortisol. Samples of salivary cortisol can reflect diurnal pulsations of cortisol (with typical increases after awakening and then decreases through the day) or acute reactivity toward stressors: cortisol typically increases about 20 min after a perceived stressor with a recovery of about 70 min later (Engert et al., 2011). At this time, our findings do not provide enough evidence to endorse one single measure of physiologic stress in children. Various biomarkers may reflect ongoing processes to support normal physiologic function, may have advantages and disadvantages in approximating stress responses, and may change along with advances in technology. For example, researchers may want to consider measuring more long‐term indices of stress responses, such as hair cortisol, with chronic stressors, such as poverty. However, there may be cases when it is important to understand a child's momentary reaction to a stressor, such as using measures of reactive salivary cortisol within a context of chronic stress, such as poverty. This may help understand how children live in poverty or may react to acute stressors within structured settings, such as in the classroom. This information could be critical to helping reduce wellness‐related disparities for children living with low income.

### 4.1 Limitations

While we approached this review in a systematic a priori fashion, some studies may have been missed. This may have been why some research on other products of the HPA axis (such as corticotropin‐releasing hormone, adrenocorticotropic hormone, and quantity of glucocorticoid receptors), sometimes seen in adult research (Miller et al., 2007), did not manifest in the search results. Additionally, some research may have been missed due to researcher degrees of freedom or publication bias, as research with significant findings tends to be published more often (“The importance of no evidence,” 2019). Another limitation of this review is that we did not conduct a meta‐analysis due to the heterogeneity of the research and findings. Yet, this is one of the first studies to concurrently examine two stress response systems in children to identify potential patterns in response to psychosocial stressors. While we attempted to synthesize the research by identifying patterns, it is possible that the heterogeneous manner of measuring psychosocial stressors and stress responses (e.g., log‐transformation of cortisol values or not, averaging morning values, calculating slopes, measuring reactivity) limited our ability to fully synthesize findings. We also did not detail moderators of the findings of the relationship between psychosocial stressors and stress responses. It may be that, for example, child age and sex and caregiver buffering could affect a child's physiological stress response to known psychosocial stressors. For example, research suggests that age may affect hair cortisol values (Bates et al., 2017; Bryson et al., 2021). Child sex may also have an impact, but it could be that sex differences in stress hormones are not yet apparent due to low prepubertal levels of sex hormones or incomplete socialization of “appropriate” gender‐specific stress responses in young children (Bates et al., 2017). Finally, one noticeable limitation of this study was that most of the children from the study samples were drawn from White, Educated, Industrialized, Rich, or Democratic (WEIRD) samples. Without including diverse individuals from non‐WEIRD contexts, the interpretation and generalizability of our findings are limited. Because many samples from developing countries were not included in this study, we will continue to have bias in our understanding of the relationship between psychosocial stressors and child stress responses. This also limits our ability to draw conclusions about potential targets for intervention.

### 4.2 Future research

Overall, the mixed findings of this review may reflect that on a population level, it may be difficult to generalize stress responses in young children without consistent measurement of the characteristics or circumstances surrounding the real‐world stressor or stress response. For example, maternal depression as a psychosocial stressor to a young child may have differential timing, duration, and severity—children may also have buffering support from a nondepressed father, which could reduce the effects of maternal depression on a child's stress response system. Given the conglomerate of characteristics that could shape relations between psychosocial stressors and stress responses in young children, researchers should consider organizing collective efforts to measure common data elements and consider additional characteristics surrounding psychosocial stressors, including moderators, to advance this area of research.

Notably, most of the studies that resulted from the search measured aspects of cortisol in saliva. Research suggests that hair cortisol measures in young children are emerging (Bates et al., 2017), as 10 years earlier, there was no mention of hair cortisol in a meta‐analysis of HPA axis stress responses in adults (Miller et al., 2007). Other trends in noninvasively estimating stress responses are also emerging, such as the cortisol content in nails (Liu & Doan, 2019). Because of the mixed findings from this study, it may be that the field is not yet able to measure stress responses precisely in young children. Because cortisol content can represent normal physiological processes, it may be that measuring cortisol as a stress response is not precise enough to capture true stress responses for more naturalistic stressors examined in this study. It may also be that children's stress responses are complex, and perhaps are buffered by unmeasured variables from these studies, which limit the detection of a stress response to certain psychosocial stressors. Another consideration is that most of the psychosocial stressors examined in this study may be considered chronic, which could affect interpretation of more acute measures of stress, such as salivary measures of cortisol, alpha‐amylase, or RSA. Moving forward, researchers should consider specific characteristics of the stressor and the child's stress responses, such as the HPA feedback circuit, type of the threat such as distant (e.g., socioeconomic status) or proximal threats (direct maternal hostility towards the child), and more nuances of the timing (e.g., pregnancy trimester) and duration of the stressor (e.g., length of untreated maternal depression).

Another consideration in advancing this research is exploring additional molecular or genomic mechanisms surrounding child stress responses to better understand how and why children differentially react to stress. For example, one included study showed that genetic variation in the child's physiologic stress response was linked to differential stress responses (Ludmer et al., 2015). This study highlights an expanding body of research on the genomic architecture of young children's developing stress responses to early psychosocial stressors. For example, epigenetic studies show that early caregiving shapes DNA methylation in stress response genes (Parent et al., 2017), leading to varied stress responses in infants (Lester et al., 2018). There is also debate and mixed findings that the methylation of stress response genes is intergenerationally transmitted (Moog et al., 2018; Ramo‐Fernández et al., 2019), suggesting that researchers perhaps consider taking a two‐generation approach in understanding offspring's stress responses. A two‐generation approach may be beneficial in understanding how methylation of stress response genes is intergenerationally transmitted and influences stress responses in offspring, as heritability of some types of stress biomarkers in children has been at least 50% (e.g., hair cortisol; Rietschel et al., 2017; Tucker‐Drob et al., 2017). A two‐generation approach may also illuminate nuances of the relationship between maternal prenatal and postnatal stress and child stress responses.

One final suggestion for future research surrounds expanding research more deeply into the family‐level context. While we focused this study on microenvironmental aspects surrounding the child's home, we did not consider other factors that could influence the child's microenvironment such as early childhood education centers or daycares, historic economic policies (e.g., family economic relief packages from COVID‐19), and neighborhood conditions on child stress responses (e.g., noise, family access to food choices). For example, some children may spend a significant amount of awake time away from their home environment, such as at an early education center. Theoretically, the time‐intensive interactions the child has with historically low‐paid (Whitebook et al., 2018) and burnt‐out teachers in the United States (Otten et al., 2019), who also have substantial turnover (Caven et al., 2021), may affect the child's stress response system. The transition to school may also be stressful (Groeneveld et al., 2013) until the child can adapt to the new environment. Further, most of the studies on parental mental health were regarding maternal mental health. It will be critical in advancing this research to understand the impact of paternal mental health and that of other caregivers on child stress responses. Finally, other environmental factors such as economic policies, neighborhood noise, and family access to food choices may deeply affect conditions of the family environment. These factors could be explored in future research as potential moderators of the child's stress response within the family context.

### 4.3 CONCLUSION

The large and expanding body of research on psychosocial stressors and stress responses in young children continues to pose more questions and answers. We used the ecobiodevelopmental framework (Shonkoff, 2010; Shonkoff et al., 2012) to guide this review. This framework proposes that the main and most proximal stressful environments to the child that contain potentially direct psychosocial stressors are associated with low levels of family resources (e.g., low parental SES, parental mental health, adverse family relationships, unsafe environments). Findings on child physiologic stress outcomes were primarily dysregulated (both blunted and increased) across diverse psychosocial stressors. Addressing the mixed results, we reviewed key findings and implications for future research.

## CONFLICT OF INTEREST

The authors declare no conflict of interest.

## Supporting information

Online Appendix infornation

## Data Availability

The data from this systematic review are directly available from published papers. The organized data used to inform this study can be made available upon request.
