# Supplementary material for: Early childhood stress responses to psychosocial stressors: The state of the science
Source: Dev Psychobiol. 2022 Sep 5;64(7):e22320. doi: 10.1002/dev.22320 (PMC9543576; doi:10.1002/dev.22320)
Supplement: Supplementary file 1 — Online Appendix infornation [file DEV-64-0-s001.docx]

**Appendix A**

**Copy of PROSPERO Protocol**

Systematic review of Hypothalamus-Pituitary-Adrenal (HPA) axis and Autonomic Nervous System (ANS) ANS physiologic responses to psychosocial stress in early childhood

Anticipated or actual start date: 3/1/2020

Anticipated completion date: 3/8/2020

**Authors**

Randi Bates, Lisa Militello, Hugo Gonzalez-Villasanti, Kammi Schmeer

**Review question**

What is the current state of the science regarding physiologic biomarkers of psychosocial stress from the hypothalamus-pituitary-adrenal (HPA) axis and autonomic nervous system (ANS) in early childhood?

S**earches**

We will search the following electronic bibliographic databases: PubMed, Web of Science, Scopus, MEDLINE, EMBASE, PsycINFO, and CINAHL. The search strategy will include only terms relating to or describing the HPA axis and ANS biomarkers of psychosocial stress in children (limited to 1-6 years of age in databases with an option to limit by age in number of years). The search terms will be adapted for use with other bibliographic databases in combination with database-specific filters, where these are available. There will be English language restrictions. The searches will be re-run just before the final analyses and relevant further studies will be included.

**URL to search strategy**

<https://docs.google.com/document/d/1HBqkXolNn9v0LZRXVbK8u3_rnj3_DLoOwR_1GFub094/edit?usp=sharing>

Example search strategy:

(hypothalamus pituitary adrenal OR autonomic nervous system) AND (child or pediatric or infant) AND (stress or distress)

**Condition or domain being studied**

HPA and ANS biomarkers of psychosocial stress in young children 1-6 years of age.

**Participants/population**

**Inclusion**: children (from 1-6 years of age) Exclusion: school-age children (children greater than 6 years of age), adolescents (13 years of age or older), adults and elderly, children with neurological disorders that physiologically impact HPA and ANS functioning

**Intervention(s), exposure(s)**

Because the use of biomarkers related to psychosocial stress in a pediatric population is still nascent, we will not be targeting a specific intervention, but will be performing an exploratory review of the literature to understand the current state of the science. Our priority is a review of the biomarkers of physiologic stress, rather than the interventions.

**Comparator(s)/control**

Because the use of biomarkers related to psychosocial stress in a pediatric population is still nascent, we will not be targeting a specific intervention, but will be performing an exploratory review of the literature to understand the current state of the science. Therefore, there are no control or comparator groups.

**Types of study to be included**

Non-experimental, quasi-experimental, or experimental studies of physiologic biomarkers of stress in early childhood populations and published through peer-review methods will be included.

**Context**

Studies involving children from 1 to 6 years of age that include ANS or HPA axis physiologic biomarkers of psychosocial stress. There are no exclusion criteria based on setting.

**Main outcome(s)**

A descriptive account of physiologic ANS and HPA axis biomarkers of psychosocial stress that are currently being reported in pediatric research.

**Timing and effect measures**

The biomarker measurement must occur between 1-6 years of age.

**Additional outcome(s)**

none

**Data extraction (selection and coding)**

All articles from the search will be compiled into a single EndNote file, where we will remove duplicates. Articles will then be uploaded to Covidence for screening. Two reviewers (RB and LKM) will screen these remaining articles based on the inclusion criteria. If discrepancies arise during the initial screening, the two reviewers will discuss until they reach a consensus. If the two reviewers are not able to reach a consensus, the third and fourth reviewers (HGV and KS) will assess the article using the inclusion criteria. Once the reviewers reach consensus, two reviewers (RB and LKM) will read the remaining articles in conjunction with the inclusion criteria to determine final inclusion. Additional discrepancies will be resolved using the above procedure. Once the reviewers have reached consensus on the articles to be included in the study, the reviewers will hand-search the reference list of each of these articles for possible inclusion of other articles. The reviewers will also locate articles that cited the included articles to determine if these articles should be included in the review. Two reviewers (RB and LKM) will screen the full-text of these articles in conjunction with the inclusion criteria to determine if the article should be included in the review. Once the final articles are determined, the two reviewers (RB and LKM) will separately extract agreed upon information from each study. Data to be extracted includes: type of study, sample demographics and characteristics, stressor exposure, biomarkers measured, biomarker source (hair, saliva, blood, other), duration of biomarker collection, and outcomes. The reviewers will use a standardized tool to assess study quality and evidence synthesis from the included studies. The reviewers will then compare the data extractions to ensure accuracy in reporting information. Discrepancies will be identified and resolved through discussion (with third and fourth authors where necessary).

**Risk of bias (quality) assessment**

Two reviewers will assess the study quality described in each full text article using the Cochrane checklists (Higgins, 2011). Given that the subject area is nascent, we will not exclude studies based on quality.

**Strategy for data synthesis**

A narrative/ descriptive synthesis is planned.

**Analysis of subgroups or subsets**

n/a

**Contact details for further information**

Randi Bates

Bates.204@osu.edu

**Organizational affiliation of the review**

The Ohio State University Crane Center for Early Childhood Research and Policy

The Ohio State University Martha S. Pitzer Center for Women, Children, & Youth

**Review team members and their organizational affiliations**

Dr. Randi A. Bates, The Ohio State University

Dr. Lisa K. Militello, The Ohio State University

Dr. Hugo Gonzalez-Villasanti, The Ohio State University

Dr. Kammi Schmeer, The Ohio State University

**Collaborators**

No others

**Type and method of review**

Systematic review

**Anticipated or actual start date**

01 January 2020

**Anticipated completion date**

31 August 2020

**Funding sources/sponsors**

N/A

**Conflicts of interest**

None

**Language**

English

**Country**

United States of America

**Stage of review**

Review Ongoing

**Subject index terms status**

Subject indexing assigned by Centre for Reviews and Dissemination.

**Subject index terms**

Biomarkers; Child; Humans; Stress, Physiological; psychosocial

**Date of registration in PROSPERO**

XX

**Date of publication of this version**

XX

**Details of any existing review of the same topic by the same authors**

Dr. Bates published a focused review on hair cortisol as a biomarker of stress in children ages 1-5 years

**Stage of review at time of this submission**

Stage Started

Preliminary searches No

Piloting of the study selection process No

Formal screening of search results against eligibility criteria No

Data extraction No

Risk of bias (quality) assessment No

Data analysis No

Versions
